# Genome-wide association study of sleep in *Drosophila melanogaster*

**DOI:** 10.1186/1471-2164-14-281

**Published:** 2013-04-25

**Authors:** Susan T Harbison, Lenovia J McCoy, Trudy FC Mackay

**Affiliations:** 1 Department of Genetics, North Carolina State University, Raleigh, North Carolina, 27695, USA; 2 Present address: Laboratory of Systems Genetics, National Heart Lung and Blood Institute, National Institutes of Health, 10 Center Dr. MSC 1654, Building 10, Room 7D13, Bethesda, MD, 20892, USA

**Keywords:** Sleep, Drosophila melanogaster, Genome-wide association, Genetic variance of environmental variation

## Abstract

**Background:**

Sleep is a highly conserved behavior, yet its duration and pattern vary extensively among species and between individuals within species. The genetic basis of natural variation in sleep remains unknown.

**Results:**

We used the *Drosophila* Genetic Reference Panel (DGRP) to perform a genome-wide association (GWA) study of sleep in *D. melanogaster*. We identified candidate single nucleotide polymorphisms (SNPs) associated with differences in the mean as well as the environmental sensitivity of sleep traits; these SNPs typically had sex-specific or sex-biased effects, and were generally located in non-coding regions. The majority of SNPs (80.3%) affecting sleep were at low frequency and had moderately large effects. Additive models incorporating multiple SNPs explained as much as 55% of the genetic variance for sleep in males and females. Many of these loci are known to interact physically and/or genetically, enabling us to place them in candidate genetic networks. We confirmed the role of seven novel loci on sleep using insertional mutagenesis and RNA interference.

**Conclusions:**

We identified many SNPs in novel loci that are potentially associated with natural variation in sleep, as well as SNPs within genes previously known to affect *Drosophila* sleep. Several of the candidate genes have human homologues that were identified in studies of human sleep, suggesting that genes affecting variation in sleep are conserved across species. Our discovery of genetic variants that influence environmental sensitivity to sleep may have a wider application to all GWA studies, because individuals with highly plastic genotypes will not have consistent phenotypes.

## Background

The need to sleep is near universal among animal species, including humans [1], yet the function of sleep remains puzzling. Sleep disorders have a devastating impact on human health. For example, individuals with narcolepsy can experience loss of muscle control and intense hallucinations during waking, which directly interferes with conscious activity [2]. Restless Leg Syndrome is a sensation of discomfort in the lower limbs during the evening that results in an inability to sleep [2]. Similarly, obstructive sleep apnea is a closing of the upper airway during sleep, restricting the brain’s access to oxygen [2]. Individuals with these sleep disorders suffer from increased daytime sleepiness and decreased cognitive performance [2,3]. Poor sleep habits as well as disordered sleep are risk factors for other diseases, such as obesity, high blood pressure, cardiovascular disease, and depression [2,4]. Sleep patterns and duration vary both among species and within species [5], due in part to segregating genetic variation [6-10], implying that sleep and risk factors for sleep disorders are at least partly under genetic control. However, the genes maintaining genetic variation in sleep are not known.

Sleep characteristics in mammals have been observed in *Drosophila melanogaster*, which facilitates the use of this powerful genetic model organism to study sleep [11,12]. *Drosophila* has several key advantages over mammals. One advantage is the extensive collections of stocks with mapped mutations, chromosomal deletions, and transgenic modifications that can be directly tested for their impact on sleep. A second key advantage is the ability to phenotype flies with identical genotypes under controlled environmental conditions, enabling the detection of environmental sensitivity and genotype-by-environment interactions. Several studies have already capitalized on community resources, together screening more than 15,000 mutations for their effects on sleep [13-15]. A small number of mutations (e.g., in *Shaker, fumin,* and *sleepless*) have major effects on sleep [13-16], but many mutations have more subtle quantitative effects [17]. A complementary approach to mutagenesis is to identify loci at which alleles with more subtle effects segregate in natural populations [18,19]. Single nucleotide polymorphisms (SNPs), insertions, and deletions in a natural population of flies are mutations that have survived the filter of natural selection and can be tested via genome-wide association (GWA) for effects on genetic variation in sleep [20]. Here, we use a new community resource, the *Drosophila* Genetic Reference Panel (DGRP) [20], to identify SNPs associated with natural variation in sleep. The DGRP is a panel of inbred lines that were created by mating full siblings of wild-caught isofemale lines for 20 generations [20]. The availability of full sequence data, the rapid decay in linkage disequilibrium (LD) with physical distance, and the lack of population structure in the DGRP [20] are advantageous conditions for a GWA study of sleep.

Using continuous sleep and activity data recordings from groups of individuals with identical genotypes, we calculated sleep duration, the number of sleep bouts or ‘naps’, and average sleep bout length during the day and night. We also measured waking activity, a measure that describes the activity level relative to the time spent awake. All parameters of sleep architecture, as well as waking activity, were genetically variable among the DGRP lines. In addition, we observed considerable variability in sleep among flies with identical genotypes, and the environmental sensitivity with respect to sleep parameters was also genetically variable. We performed GWA analyses for the mean and environmental sensitivity of each sleep trait in the DGRP, and found many SNPs individually associated with each sleep trait, often with sex-specific effects. Most (80.3%) SNPs associated with the mean and environmental variance of sleep had relatively low minor allele frequencies (0.024 - 0.05). Additive multi-SNP models revealed that 1–4 SNPs could explain 19.5-55.5% of the genetic variance in sleep for females, and 18.0-55.6% of the genetic variance in sleep for males. While further work is required to identify causal variants, we found that individual SNPs associated with sleep traits were located in genes known to have effects on sleep in flies, in genes over-represented in the *Epidermal growth factor receptor* (*Egfr*) pathway, and in genes with homologs previously implicated in human sleep. Low-frequency alleles were associated with increased environmental sensitivity. Further, genes associated with sleep had signatures of purifying selection [20]. These observations raise the intriguing possibility that sleep may be canalized in heterogeneous natural populations [21].

## Results

### Quantitative genetic analyses

**Figure 1:**
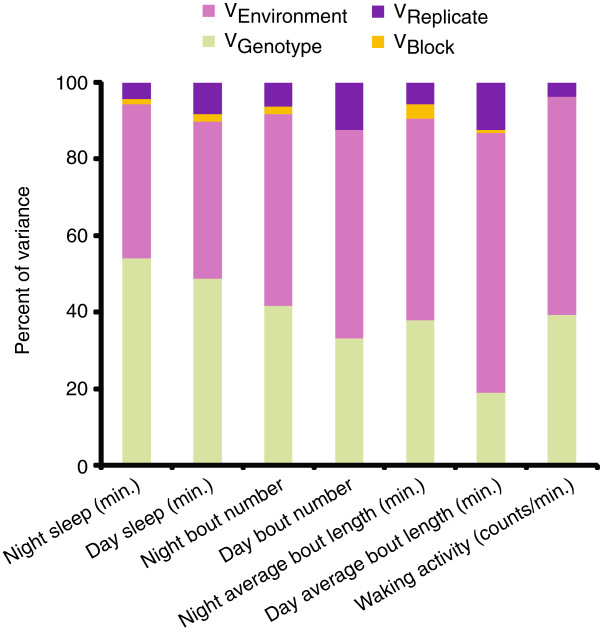
Partitioning of variance in sleep in the DGRP lines.

**Figure 2:**
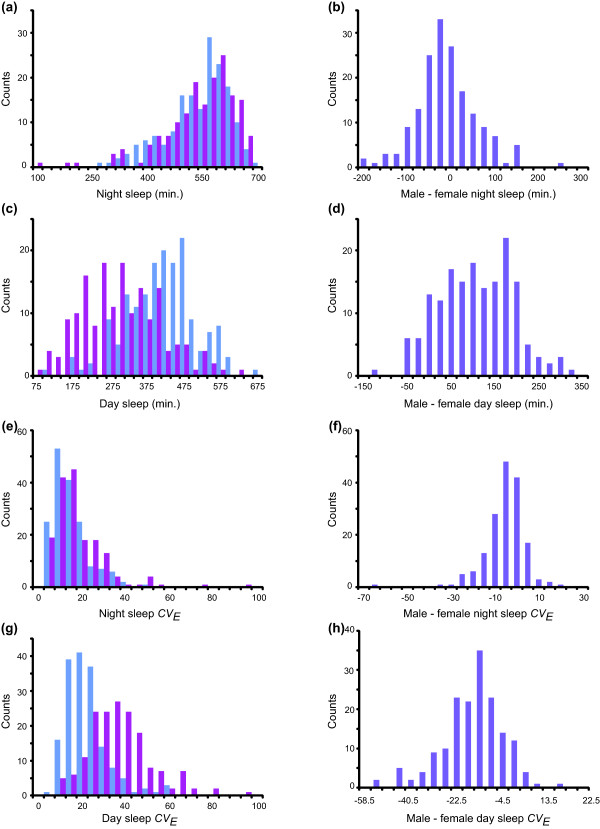
**Histograms of mean and the coefficient of environmental variation (*CV***_***E***_**) for sleep duration.** Blue bars denote male line means, pink bars denote female line means, and the difference in line means between males and females (male – female) is shown by purple bars. (**a**) Night sleep. (**b**) Male - female night sleep. (**c**) Day sleep. (**d**) Male – female day sleep. (**e**) Night sleep *CV*_*E*_. (**f**) Male – female night sleep *CV*_*E*_. (**g**) Day sleep *CV*_*E*_. (**h**) Male – female day sleep *CV*_*E*_.

We measured day and night sleep duration, average bout length, bout number and waking activity, separately for males and females, in 168 DGRP lines. Quantitative genetic analyses of each trait revealed substantial and highly significant genetic variation among the lines for the mean of each trait (Figure 1, Additional file 1). Estimates of broad sense heritability (*H*^2^) for night sleep, day sleep, night bout number, day bout number, night average bout length, day average bout length, and waking activity, were, respectively *H*^2^ = 0.54, *H*^2^ = 0.49, *H*^2^ = 0.42, *H*^2^ = 0.33, *H*^2^ = 0.38, *H*^2^ = 0.19 and *H*^2^ = 0.39 (Additional file 1). All sleep traits except for night average bout length and night sleep duration were highly sexually dimorphic (Figure 2, Additional files 1 and 2). However, for all traits there was substantial genetic variation in sexual dimorphism (i.e., the difference in sleep traits between males and females varied among the lines), as shown by differences in genetic variance between females and males for most traits; significant sex by line interaction terms; and cross-sex genetic correlations (*r*_*MF*_) ranging from a low of *r*_*MF*_ = 0.58 for day bout number to a high of *r*_*MF*_ = 0.84 for waking activity (Figure 2, Additional files 1 and 2). Thus we expect that some polymorphisms will affect sleep in both sexes, while others will have sex-specific or sex-biased effects. The range of variation in sleep traits encompassed by the DGRP lines is astonishing (Figure 2, Additional file 2), with mean sleep duration spanning much of the possible 24-hour spectrum. Night sleep times ranged from 122 to 689 minutes in females and from 278 to 703 minutes in males (Figure 2a); females tended to sleep slightly longer during the night than males (Figure 2b). Day sleep duration ranged from 94 to 640 minutes in females and from 120 to 682 minutes in males (Figure 2c). As previously noted in other natural populations of flies [7,22] males generally slept more during the day than females (Figure 2d).

**Figure 3:**
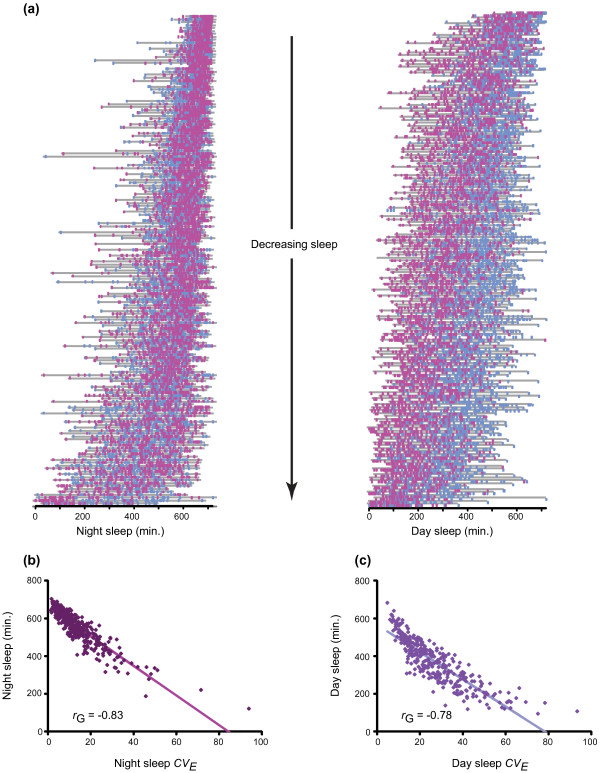
**Genotypic influences on individual variability in sleep duration.** (**a**) Night and day sleep duration for every fly in the experiment, segregated by genotype and ordered from shortest-sleeping to longest-sleeping line. Male flies are represented by blue dots; females are represented by pink dots. Gray horizontal bars show the range of sleep duration in each line. (**b**) and (**c**) Plot of sleep duration line mean versus sleep duration *CV*_*E*_. (**b**) Night sleep. (**c**) Day sleep.

Sleep was highly variable among individual flies with identical genotypes (Figure 3a), and most variable in lines having the shortest mean sleep times. We calculated the environmental coefficient of variation (*CV*_*E*_) for all sleep parameters to determine whether there was genetic variation in the magnitude of environmental sensitivity; *i.e.*, whether some lines are relatively more canalized and others more phenotypically plastic in response to the same random environmental effects [23]. Here, the *CV*_*E*_ for sleep between individuals can be estimated directly for replicates of each line as flies from a given line in the DGRP have identical genotypes (see Methods). We partitioned the variance in *CV*_*E*_ between and within lines (Additional file 1). We found that *CV*_*E*_ had a significant genetic component for all sleep traits, with *H*^2^ = 0.72 for night sleep duration *CV*_*E*_, *H*^2^ = 0.55 for day sleep duration *CV*_*E*_, *H*^2^ = 0.31 for night bout number *CV*_*E*_, *H*^2^ = 0.47 for day bout number *CV*_*E*_, *H*^2^ = 0.15 for night average bout length *CV*_*E*_, *H*^2^ = 0.06 for day average bout length *CV*_*E*_, and *H*^2^ = 0.24 for waking activity *CV*_*E*_ (Additional file 1). All traits but waking activity exhibited sexual dimorphism for *CV*_*E*_, with generally greater sensitivity to environmental variation in females than males (Figures 2e- 2h; Additional files 1 and 2). There was genetic variation in the magnitude of *CV*_*E*_ between males and females for night sleep, day sleep, night bout number, and day bout number, with cross-sex genetic correlation estimates of *r*_*MF*_ = 0.74, *r*_*MF*_ = 0.61, *r*_*MF*_ = 0.71 and *r*_*MF*_ = 0.38, respectively (Additional file 1). Thus, we expect to be able to identify genetic variants affecting environmental sensitivity for sleep traits, and some of these variants will be the same for males and females, while others will be sex-specific or sex-biased.

We computed genetic correlations between lines for the mean value and *CV*_*E*_ of each sleep trait to assess the extent to which the same variants affect both the mean and environmental sensitivity. There were strong negative genetic correlations (*r*_*G*_) between night and day sleep duration and *CV*_*E*_ (*r*_*G*_ = −0.83 for night sleep duration and *r*_*G*_ = −0.78 for day sleep duration) (Figures 3b and 3c). For these traits, we expect largely the same variants to affect the mean and environmental sensitivity. This pattern held for most, but not all, sleep traits. The correlations were *r*_*G*_ = −0.75 for night bout number, *r*_*G*_ = −0.51 for day bout number, *r*_*G*_ = −0.08 for night average bout length, *r*_*G*_ = 0.86 for day average bout length, and *r*_*G*_ = 0.33 for waking activity (Additional file 1). The low *r*_G_ for night average bout length implies that variants affecting the mean do not affect environmental sensitivity, and *vice versa*; however, the lower genetic correlations are also in part due to lower heritabilities of both mean and sensitivity. Mean phenotypic values of all sleep parameters in the DGRP lines are given in Additional file 3.

### Genotype-phenotype associations

**Figure 4:**
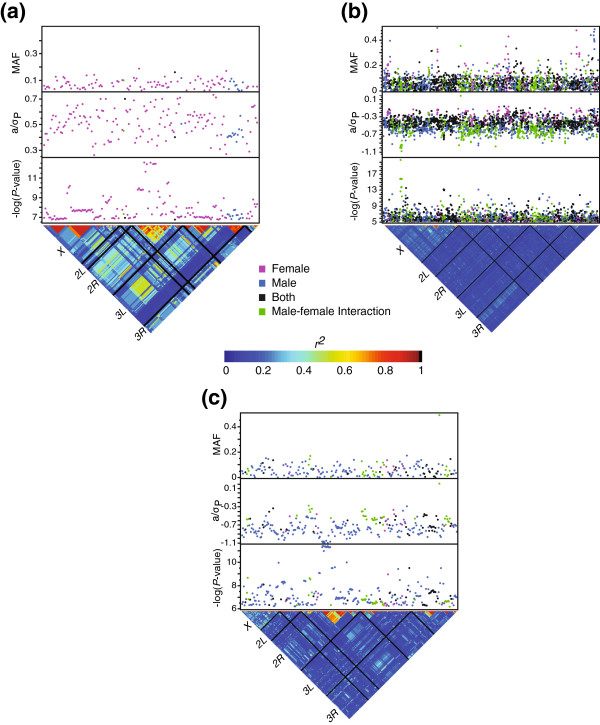
**Genome wide association results for mean sleep traits.** Significant SNPs (FDR ≤ 0.01) are plotted. The top panel shows the minor allele frequency (MAF) for each significant SNP. Effect sizes normalized by the phenotypic standard deviation (*a*/*σ*_*P*_) are plotted in the middle panel. *P*-values are plotted as –log_10_(*P-*value) in the bottom panel. The lower triangle shows the distribution of linkage disequilibrium among SNPs as *r*^*2*^. Solid black lines identify the five major chromosome arms. (**a**) Night sleep. (**b**) Day average bout length. (**c**) Waking activity.

**Figure 5:**
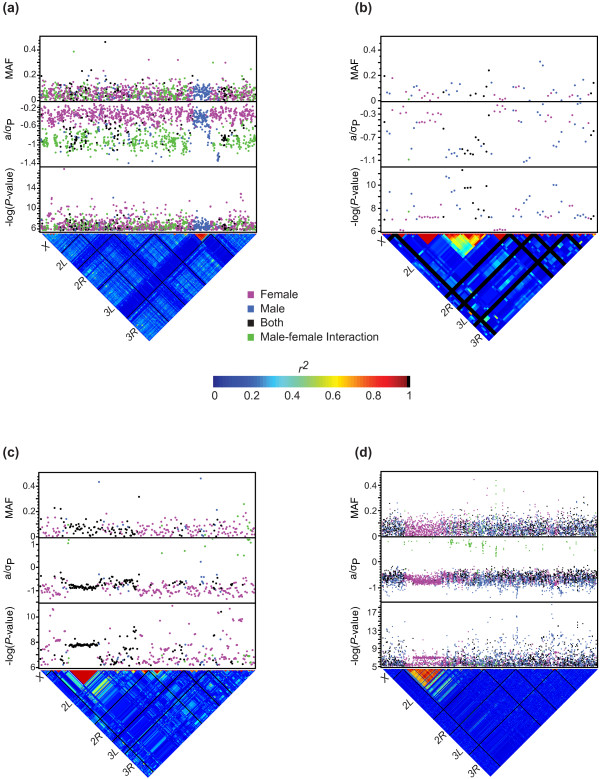
**Genome wide association results for sleep *CV***_***E ***_**traits.** Significant SNPs (FDR ≤ 0.01) are plotted. The top panel shows the minor allele frequency (MAF) for each significant SNP. Effect sizes normalized by the phenotypic standard deviation (*a*/*σ*_*P*_) are plotted in the middle panel. *P*-values are plotted as –log_10_(*P*-value) in the bottom panel. The lower triangle shows the distribution of linkage disequilibrium among SNPs as *r*^*2*^. Solid black lines identify the five major chromosome arms. (**a**) Night sleep *CV*_*E*_. (**b**) Day sleep *CV*_*E*_. (**c**) Day bout number *CV*_*E*_. (**d**) Waking activity *CV*_*E*_.

The DGRP lines have been sequenced [20]. We used 2,490,165 SNPs for which the minor allele is present in at least four lines to conduct GWA analyses for the mean and *CV*_*E*_ of all sleep traits. With this number of SNPs there is a distinct possibility of many false positives; we therefore limited our significance threshold to a false discovery rate (FDR) of 0.01 or lower for each sleep trait. We found many SNPs associated with the mean and coefficient of environmental variation of most sleep traits using this threshold (Table 1). The exceptions were day sleep and night bout number, for which none of the associations met this threshold. As has been observed in human studies [24], many SNPs were located in intergenic regions (49.1%) and in introns (35.3%), while fewer SNPs were found in coding sequences (15.5%), roughly corresponding to the amount of DNA in the genome for these three categories (Additional file 4) [25]. Consistent with the high genetic correlation between the mean and environmental variance for sleep duration, 95.6% of the SNPs overlapped between night sleep mean and *CV*_*E*_ (Table 1). As expected from the quantitative genetic analyses of sleep phenotypes, the SNPs significantly associated with sleep were often sex-specific or sex-biased. We classified SNPs as sex-specific (significant in one sex only), sex-biased (significant for both sexes, but with greater effects in one sex versus the other), or sex-antagonistic (significant for both sexes, but with the effects on sleep occurring in opposite directions) [26]. The overwhelming majority of SNPs exhibited some degree of sex dimorphism: 27.7% percent of the SNPs were sex-specific, 51.3% were sex-biased, and 1.8% were sex-antagonistic, with the remaining 19.2% affecting both sexes equally (Figures 4 and 5). Quantile-quantile (Q-Q) plots for each sleep trait show *P*-values deviating from the expected distribution for some of the sleep traits. We therefore investigated whether sleep was influenced by population structure due to cryptic relatedness in the DGRP. Correcting the association tests for relatedness resulted in no appreciable differences in the Q-Q plots (Additional files 5 and 6) or distributions of *P*-values (Additional files 7 and 8), suggesting that the deviations are not caused by population admixture. Although on average LD decays rapidly with physical distance in the DGRP [20], there is great variation around this average decay, such that there are some regions of local LD, including LD associated with polymorphic inversions. We speculated that this local LD could cause the appearance of structure in the Q-Q plots. We therefore assessed whether the presence or absence of common inversions in the DGRP (M. A. Carbone, A. Yamamoto, Y. Inoue, and T. F. C. Mackay, personal communication) were associated with sleep traits. Indeed, day average bout length, day average bout length *CV*_*E*_, and waking activity *CV*_*E*_ were significantly associated with the *ln_3R_Mo* (Missouri) inversion; day average bout length, day bout number, and day average bout length *CV*_*E*_ were associated with the *ln_2R_NS* (Nova Scotia) inversion; and day sleep, day sleep *CV*_*E,*_ night bout *CV*_*E*_, and day bout *CV*_*E*_ were associated with the *ln_2L_t* inversion. We also observed many instances of local LD independent of the inversions. For night sleep, the largest regions of LD were a ~10,277 kb region of LD from the intronic region of *CG11085* to the intronic region of *fog*, and a smaller 702 bp region just forward of and within *CG14431* on the *X* chromosome; a ~6,380 kb region of LD between an intergenic region forward of *CG12523* and within the intronic region of *Rbp6* on Chromosome *3L*; and LD between almost exclusively male-specific SNPs in a ~345 kb region spanning the intergenic region between *α Catenin* and *Argonaute 3*, as well as within *Argonaute 3* introns for both night sleep and night sleep *CV*_*E*_ on *3L* (Figures 4a and 5a). There were no large LD regions associated with day average bout length (Figure 4b), but many small regions were identified. For waking activity, we observed LD between SNPs in a ~2,569 kb region on chromosome *2R* between SNPs in the coding region of *CG16868* and the region forward of *RpL23* (Figure 4c). For day sleep *CV*_*E*_, we observed LD in a region surrounding and including *ms(2)34Fe* on chromosome *2L* (Figure 5b). It is not possible to distinguish causal SNPs within chromosomal inversions and regions of localized LD. Furthermore, it is not possible to incorporate the effects of the inversions in the GWA model as the genotype of each SNP is confounded with the presence or the absence of the inversion. We therefore designated one SNP as a proxy for all the significant SNPs found in a given chromosomal inversion or region of LD for the purpose of tallying the total number of significant SNPs, which reduced the total number of significant SNPs by 18-40% (Table 1). Additional files 9 and 10 provide detailed information on each significant SNP in this study, including the classification of SNPs within LD blocks and chromosomal inversions.

**Table 1:** Results of the genome wide association of sleep parameters and SNPs from the DGRP

**Trait**	Trait mean SNPs^*^	% Low frequency Alleles^**^	**Trait*CV***_***E***_SNPs^*^	% Low frequency Alleles^**^	**Overlapping SNPs (%)**	**Trait mean genes**	**Trait*CV***_***E***_**genes**	**Overlapping genes**
Night sleep	160 (120)	87.5	1,552 (1,175)	82.0	95.6	53	456	52
Day sleep	0 (0)	0	71 (51)	76.0	0.0	0	23	0
Night bout no.	0 (0)	0	3 (3)	33.3	0.0	0	0	0
Day bout no.	3 (3)	0	340 (198)	82.1	0.0	2	103	0
Night average bout length	16 (16)	68.8	1 (1)	100.0	0.0	6	1	0
Day average bout length	2,082 (1,174)	76.7	5 (3)	100.0	0.1	551	3	2
Waking activity	264 (214)	89.4	4,083 (2,727)	80.7	10.6	87	970	45

^*^Numbers of SNPs listed have FDR ≤ 0.01; numbers of SNPs accounting for inversions and local LD blocks are listed in parentheses. See text for additional detail.

^**^ Low frequency is defined here as 0.024 ≤ minor allele frequency ≤ 0.05.

Most SNPs associated with sleep trait means and *CV*_*E*_ were at the low range of the allele frequency spectrum, with minor allele frequencies between 0.024 and 0.05 (Table 1). Low frequency alleles were associated with short night sleep duration, long day average bout length, and increased waking activity. Interestingly, the lines most sensitive to random environmental perturbations, i.e., the lines with the highest *CV*_*E*_, had a preponderance of low-frequency alleles; this was a feature of the *CV*_*E*_ for all sleep traits. As anticipated, low minor allele frequencies were associated with larger effect sizes for all sleep traits (Additional file 11), and effect sizes for each SNP were large (Additional files 9 and 10). The genetic variance (*V*_*G*_) explained by each SNP in a population of inbred lines is *V*_*G*_ = 4*pqa*^2^, where *p* and *q* are the frequencies of the major and minor alleles, and *a* is one half of the difference in mean between lines bearing the major and minor alleles [27]. Summing the genetic variance over all SNPs overestimates the total genetic variance. This is because SNPs are not totally independent due to chromosomal inversions and residual LD (Figures 4 and 5; Additional files 9 and 10); the threshold chosen to minimize false negative associations will include variable numbers of false positive associations; and the truncated distribution of effect sizes, in addition to sample size considerations, leads to an overestimation of the amount of variance explained known as the Beavis effect [28]. We therefore performed iterative GWA analyses that estimated the additive effects of multiple SNPs simultaneously. We found that 1–4 SNPs could be used to explain 19.5-55.5% of the genetic variance in sleep in females, and 18.0-55.6% of the genetic variance in sleep in males (Additional file 12). Roughly half (54.4%) of the SNPs we found using the additive multi-SNP model were also significant as a single SNP; the remainder only appear when considered as part of the multi-SNP model.

Further experimental work is required in order to definitively identify causal SNPs, particularly those within inversions or regions of local LD. However, we note that many of the SNPs are in plausible candidate genes, including 19 genes in which mutations were previously associated with *Drosophila* sleep (Additional file 13), and 10 genes for which the human ortholog has been associated with sleep and sleep disorders in humans, nine of which are not in LD with other genes (*CG31646*, *dnc*, *Dscam*, *eya*, *l(3)82Fd*, *nudE*, *ppan*, and *trbl*) and one (*Lar*) that is in LD with other potential candidate genes (Table 2). Of the 1,300 genes associated with one or more sleep traits, 213 are associated with potentially functional missense or nonsense mutations [20]; 757 are expressed in the adult brain and 764 are expressed in the larval CNS [29]. The coding sequence of most genes associated with sleep (419) appeared to be under purifying selection, consistent with previous observations [7], but some genes (17) were rapidly evolving [20]. Several (10) genes were previously identified as quantitative trait transcripts associated with natural variation in sleep in 40 DGRP lines [7]; and 320 genes, although previously not implicated in sleep, are associated with SNPs with *P*-values exceeding a Bonferroni correction (*P* < 2 × 10^-8^) for multiple tests.

**Table 2:** Candidate genes from this study with human homologues implicated in normal and disordered sleep

**Human sleep trait or disorder**	**Human gene**	**Sleep trait**	***D. melanogaster*gene**	**References**
Daytime sleepiness	*PDE4D*	Day average bout length	*dunce* (*dnc*)	[43]
		Night sleep *CV*_*E*_		
		Waking activity *CV*_*E*_		
	*EYA1*	Day average bout length	*eyes absent* (*eya*)	[43]
		Day bout number *CV*_*E*_		
Usual bedtime	*OPCML*	Day average bout length	*CG31646*	[43]
		Night sleep *CV*_*E*_		
		Waking activity *CV*_*E*_		
Sleep duration	*NCOA7*	Waking activity	*l(3)82Fd*	[43]
		Night sleep *CV*_*E*_		
		Waking activity *CV*_*E*_		
Narcolepsy	*P2RY11*	Day sleep *CV*_*E*_	*peter pan* (*ppan*)	[44]
	*TRIB2*	Day bout number *CV*_*E*_	*tribbles* (*trbl*)	[45-47]
				[48]
	*Dscam*	Day average bout length	*Down syndrome cell adhesion molecule* (*Dscam*)	[49]
Restless Leg Syndrome	*MEIS1*	Waking activity	*homothorax* (*hth*)	[50]
		Waking activity *CV*_*E*_		
	*PTPRD*	Waking activity *CV*_*E*_	*Leukocyte-antigen-related-like* (*Lar*)	[51]
Sleep Apnea	*ApoE*	Night sleep	*nudE*	[52]
		Day average bout length		
		Night sleep *CV*_*E*_		

**Figure 6:**
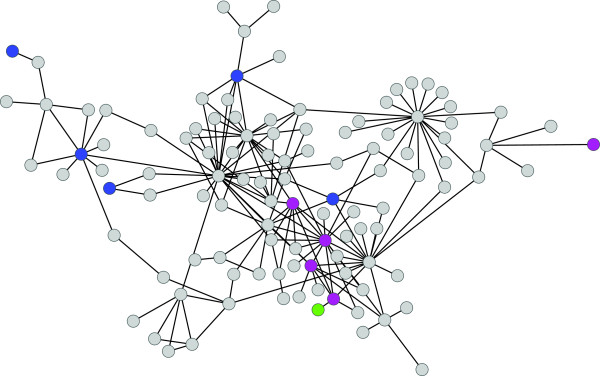
**Inferred sleep network.** Pink denotes genes that are part of the dorso-ventral axis formation (*Egfr*) pathway (*Egfr*, *rho*, *S*, *drk*, and *phl*); blue denotes genes tested in this study (*brk*, *fz*, *Hey*, *scrib*, *tkv*, and *Ubx*); green denotes *bun*, a gene previously identified as a candidate gene for sleep in *D. melanogaster*[39].

We hypothesized that the list of genes associated with SNPs affecting one or more sleep traits would be enriched for causality. If so, groups of genes should be enriched for particular Gene Ontology (GO) terms or pathways [30]. We found 1,126 GO terms that exceeded a *P*-value of 0.05 (Additional file 14). Genes involved in developmental processes of all kinds were a common theme for all sleep traits, including nervous system development and axonogenesis. Further, we found significant enrichment for genes associated with the *Epidermal growth factor receptor* (*Egfr*) pathway (*P* = 0.015). *Egfr* signaling has recently been implicated in *D. melanogaster* sleep in an independent study [31]. We used the BIOGRID data base of genes with known physical and genetic interactions [32] to query our SNP list for genes that may interact with *Egfr*. A total of 114 genes implicated by the sleep GWA analyses formed a candidate interaction network with genes from the *Egfr* pathway (Figure 6, Additional file 15).

### Functional tests

**Figure 7:**
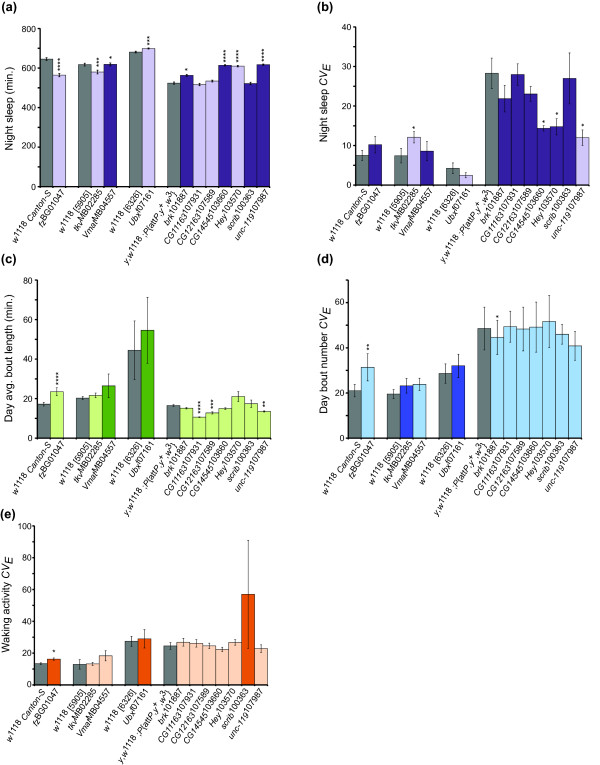
**Results of mutant and RNAi knockdown tests for night sleep parameters.** Results are grouped with their respective isogenic control, shown in gray. Combined-sex data are shown. Genes that were identified in the GWAS for a given trait are in the darker shade; genes not significant in the GWAS for a given trait are in the lighter shade. (**a**) Night sleep. (**b**) Night sleep *CV*_*E*_. (**c**) Day average bout length. (**d**) Day bout number *CV*_*E*_. (**e**) Waking activity *CV*_*E*_. ns, not significant; * 0.01 ≤ *P* < 0.05; ** 0.001 ≤ *P* < 0.01; *** 0.0001 ≤ *P* < 0.001; **** *P* <0.0001.

We selected mutations and RNAi knockdown constructs in eleven genes to evaluate effects on the mean and *CV*_*E*_ of selected sleep traits, using the criteria that the corresponding SNPs had high statistical significance in the GWA analyses (Additional files 9 and 10), the genes were ‘hubs’ in the *Egfr* interaction network (Figure 6), and the mutant or RNAi alleles were available from *Drosophila* stock collections with co-isogenic controls. We tested *P*-element insertions in *frizzled* (*fz*), *thickveins* (*tkv*), *Ultrabithorax* (*Ubx*), and *Vesicular monoamine transporter* (*Vmat*) for their effects on sleep. We also tested the effect of pan-neuronal knockdown of gene expression on sleep using RNAi constructs in candidate genes for sleep. We used an *elav*-GAL4 driver to reduce the expression of these genes in all neurons (see Methods). We tested RNAi constructs in *brinker* (*brk*), *CG11163*, *CG12163*, *CG14545*, *Hairy/E(spl)-related with YRPW motif* (*Hey*), *scribbled* (*scrib*), and *unc-119*. *Vmat*, *brk*, *CG14545*, *scrib*, and *unc-119* were implicated in night sleep. Many significant SNPS were present in *Vmat* introns and the 3^′^-UTR; these SNPs were significant for males, females, sexes pooled, and SNP × sex interactions. *brk* and *CG14545* had at least one SNP in the coding region; these SNPs were significant for sexes pooled and females separately in the GWAS. Many SNPs significant for females as well as both sexes pooled were located in the intergenic region of *unc-119*, but this gene is nested within an intron of a larger gene, *CG1677*; the SNPs in *scrib* were also intronic. Tests of the *Vmat* mutation for night sleep revealed a significant genotype × sex interaction, partially confirming the GWAS observation. The putative RNA reduction in *brk*, *CG14545* and *unc-119* gene expression resulted in significant differences from the control in females and averaged over both sexes, confirming the GWAS results. However night sleep in *scrib* was not significantly different from the control (Figure 7a and Additional file 16). We tested two mutations (*fz* and *Vmat*) and seven RNAi constructs (*brk*, *CG11163*, *CG12163*, *CG14545*, *Hey*, *scrib*, and *unc-119*) for their impact on night sleep *CV*_*E*_. A single SNP in an intron of *fz* was significant for females only in the GWAS. *CG11163*, *CG12163* and *Hey* had SNPs significant for both sexes in the genome-wide association that were located in the 5^′^-UTR, the 3^′^-UTR, and in an intron, respectively. Only the *CG14545* and *Hey* RNAi constructs had effects on night sleep *CV*_*E*_ that were consistent with the GWAS analysis (Figure 7b and Additional file 16). We also evaluated the effect of mutations on day average bout length. In the GWAS, a female-specific SNP in the 3^′^-UTR of *Vmat* and intronic SNPs in *Ubx* were implicated in day average bout length; however, mutations in these genes failed to replicate these findings (Figure 7c). We tested mutations in *tkv* and *Ubx* for their impact on day bout number *CV*_*E*_ as SNPs in introns of these genes were significant for both sexes, but we did not find any change in phenotype due to these mutations (Figure 7d). Finally, we tested three mutations (*fz*, *tkv*, and *Ubx*) and one RNAi construct (*scrib*) for their effect on waking activity *CV*_*E*_. The mutation in *fz* was significantly different from the control for sexes combined, replicating the GWAS finding (Figure 7e and Additional file 16).

Although we specifically tested the mutations and RNAi constructs for the effects outlined above, we observed significant effects on other sleep phenotypes as well. In fact, every gene tested exhibited pleiotropy (Figure 7, Additional files 16 and 17). We observed pleiotropic effects in night sleep (*fz*, *tkv*, *Ubx* and *Hey*); night sleep *CV*_*E*_ (*tkv* and *unc-119*); day average bout length (*fz*, *CG11163*, *CG12163* and *unc-119*); and day bout number *CV*_*E*_ (*fz* and *brk*). In general, the pleiotropy occurred in sleep phenotypes having some degree of genetic correlation with the trait of interest (Additional files 1 and 17). These confirmatory tests demonstrate that candidate genes impacting both mean and *CV*_*E*_ sleep phenotypes can be identified from their respective SNP associations, and mutations in these genes can also influence these traits as well as other correlated traits (Additional files 16 and 17).

## Discussion

The DGRP is a new community resource for GWA analysis of complex traits [20]. The DGRP lines harbor most common variants and a representative sample of rare variants that have survived natural selection, and the ~2.5 million SNPs interrogated in GWA studies represent novel, subtle variants that are unlikely to be produced by mutagenesis screens. We found substantial genetic variation for sleep traits in the DGRP, as has been observed previously in natural populations of flies [7,22]. A prominent feature of the genetic architecture of naturally occurring variation for all sleep traits is variation in sex dimorphism among the lines. Using a conservative FDR threshold of 0.01, we found a total of 2,427 unique SNPs associated with night sleep duration, day bout number, day and night average bout length, and waking activity. Some SNPs were in genomic regions containing chromosomal inversions or local LD; consequently, we could not distinguish among candidate SNPs in these regions. Defining a proxy SNP for those SNPs within inversions or LD groups reduced the putative number of SNPs to 2,233 SNPs. Consistent with the observation of genetic variation in sex dimorphism, the majority of SNPs had effects that were either specific to one sex or sex-biased. A few SNPs were sex-antagonistic, *i.e.*, with opposite effects in males and females. Interestingly, some SNPs were located within the canonical sex determination gene *fruitless*. However, the mechanism underlying sex-specific or sex-biased SNP effects on sleep phenotypes is unknown.

Variation in quantitative traits is traditionally partitioned into genetic (*V*_*G*_) and environmental (*V*_*E*_) variance, where the environmental variance reflects variation among individuals due to non-genetic causes [27]. In experimental populations reared under constant environmental conditions, *V*_*E*_ reflects variation among individuals due to sensitivity, or plasticity, of alleles to intangible environmental variance. Evidence has been accumulating recently that there is often genetic variation in environmental sensitivity [23]; *i.e.*, different genotypes exhibit more or less variation in the face of minor environmental perturbations [33-35]. If environmental variation is at least partially under genetic control, then trait values will be inconsistent for highly sensitive genotypes [36]. It would not be sufficient, therefore, to know only those variants that are risk alleles for the mean phenotypes used in diagnosis because one could not accurately predict which individuals would be afflicted. Environmental sensitivity may explain in part why some risk alleles are present in *unaffected* individuals [37]. Currently, the genetic architecture of genetic variation in environmental variation is not known. Do the same variants that affect the trait mean also affect differences in environmental variation, or is genetic variation in the mean and environmental variance uncorrelated? The DGRP population of inbred lines derived from wild-caught flies is an ideal scenario for estimating the extent to which there is genetic variation in environmental plasticity and mapping genetic variants associated with these traits. We found substantial genetic variance in environmental variance, measured as the coefficient of environmental variance, *CV*_*E*_, to correct for any correlation between the mean and variance within a genotype.

We found 5,963 SNPs associated with *CV*_*E*_ of sleep traits; 3,077 of which were in intergenic regions and 2,886 in an annotated gene. Although the trait mean was not correlated with *CV*_*E*_ for night average bout length, all other sleep traits were correlated with their respective *CV*_*E*_ to some degree, and in several cases, the correlation between trait mean and *CV*_*E*_ was strong. For example, nearly all of the SNPs associated with mean night sleep duration (95.6%) were also associated with *CV*_*E*_ of night sleep. Flies with short-sleeping genotypes tend to have higher *CV*_*E*_ and are thus more sensitive to changes in the micro-environment, making short sleep an inherently less stable phenotype than long sleep. This apparent instability has also been observed in severe short-sleeping mutants of *D. melanogaster*, where genetic modifiers increasing sleep tend to accumulate [13,38]. For all sleep traits, low-frequency alleles were associated with genotypes having higher *CV*_*E*_, *i. e.*, higher sensitivity, and common alleles were associated with more canalized genotypes. This observation suggests that if low frequency alleles are deleterious, they could ‘escape’ natural selection because some individuals with low frequency alleles will have the same phenotype as those bearing the common allele.

The majority of loci implicated to affect sleep in our analysis of natural variants are novel, many are in non-coding, intergenic regions, and several SNPs could potentially affect more than one gene. In total, we found 1,237 genes associated with one or more mean or *CV*_*E*_ sleep traits, including 19 genes previously associated with sleep in *D. melanogaster*, such as *Epidermal growth factor receptor*[31], *bunched*[39], *Pigment-dispersing factor receptor*[40], *rhomboid*[31], *Shaker*[13], and *Syndecan*[41] (Additional file 13). We searched the NCBI HomoloGene (Release 65) data base [42] to assess whether candidate genes from this study had homologues of genes previously implicated in human sleep studies, and identified 10 genes with human homologues affecting normal sleep characteristics as well as the sleep disorders narcolepsy, Restless Leg Syndrome (RLS), and sleep apnea (Table 2). A single human study has associated SNPs in the Framingham Heart Study Offspring Cohort with normal sleep characteristics including daytime sleepiness, usual bedtime, and sleep duration. Daytime sleepiness, a subjective assessment made by study participants using the Epworth Sleepiness Scale, was associated with several SNPs, including a SNP in an intron of *phosphodiesterase 4D* (*PDE4D*) and one in the intron of *eyes absent 1* (*EYA1*) [43]. In our study, the fly homologs of these genes, *dunce* and *eyes absent*, were also associated with sleep (Table 2). A non-synonymous SNP in *neuropeptide S receptor 1* (*NPSR1*) was associated with usual bedtime, an indicator of diurnal preference, in the human study [43]. We found SNPs in the fly homolog of this gene, *CG31646*. Sleep duration was associated with *nuclear receptor coactivator 7* (*NCOA7*) in humans [43], and its fly homolog *l(3)82Fd* was associated with night sleep and waking activity in flies.

We also found homologs of human candidate genes for sleep disorders. Narcolepsy is a chronic condition characterized by intense sleepiness during the day and disturbed sleep patterns at night. In some cases, individuals also experience cataplexy, a sudden loss of muscle control. Narcolepsy may be the result of the immune system attacking its own hypocretin neurons, as several loci associated with narcolepsy are involved in immune system function, such as the *purinergic receptor P2Y11* (*P2RY11*) gene [44]. Further, antibodies of *tribbles homolog 2* (*TRIB2*) were increased in the blood of narcoleptic patients [45-47], although their presence may not be causal [48]. Linkage mapping of a large family identified a region that includes *Down syndrome cell adhesion molecule* (*Dscam*) precursor in narcolepsy [49]. Fly homologues of these human genes, *peter pan*, *tribbles*, and *Dscam*, had significant SNPs associated with sleep phenotypes (Table 2).

We also found SNPs associated with fly sleep in three genes that are homologous to candidate genes identified for Restless Leg Syndrome (RLS), a neurological disorder distinguished by pronounced discomfort in the lower limbs that leads to disturbed sleep patterns. Loci implicated in RLS GWA studies include the developmental gene *MEIS homeobox 1* (*MEIS1*) [50] and *Protein tyrosine phosphatase receptor type D* (*PTPRD*) [51]; we found significant SNPs in their fly counterparts - *homothorax* and *Leukocyte-antigen-related-like*, respectively, though *Lar* is located in a block of local LD (Table 2).

Like narcolepsy, obstructive sleep apnea results in daytime sleepiness; but it is the result of closure of the upper airway during sleep, blocking oxygen intake for short periods and thereby disrupting sleep. *apolipoprotein E* (*APOE*) is associated with sleep apnea in a context-dependent manner, with the association stronger in younger individuals, and those with cardiovascular disease or hypertension [52]. The fly homolog of *APOE*, *nudE*, was implicated in our study.

While the DGRP fly lines do not represent models of these human sleep disorders, it is remarkable that many homologues of candidate genes for human sleep as well as sleep disorders emerge in this association study of endogenous sleep phenotypes in flies. If one assumes that human sleep disorders are the result of mis-regulation of normal sleep genes, this observation suggests that the role of these genes in sleep may be conserved across species [53].

We identified a network of 114 genes associated with sleep phenotypes in the DGRP that are known to interact either genetically or physically with *Egfr* (Figure 6, Additional file 15). Prior analyses have demonstrated effects of a few of these genes on adult sleep in *Drosophila*[31,39]; therefore the network generates testable hypotheses. We tested for altered sleep phenotypes in six novel candidate genes from the network: *brk*, *fz*, *Hey*, *scrib*, *tkv*, and *Ubx*, all of which have been previously studied for their role in development. We demonstrated that a *P*-element insertion in *fz* and pan-neuronal reduction in gene expression in *brk* and *Hey* recapitulated the GWAS results. *fz* is involved in synapse development and in planar cell polarity [54]. *brk* is a DNA-binding protein that can act to repress genes that are the targets of *Decapentaplegic* signaling [55]. *Hey* is a transcription factor that is a target of *Notch* signaling in neuroblasts, and exhibits *Notch*-independent expression in precursor neurons of the mushroom bodies as well [56]. That these genes, along with many of the genes identified in this GWAS, have a role in neural development and cellular morphogenesis begs the question of whether sleep behavior is partly the result of neural architecture, or whether the genes have an additional role in the adult fly. The GWAS results suggest that biological processes in addition to neural development play a role in sleep, however. We tested genes affecting other biological processes, as well as three genes with unknown function (CG14545, CG11163, and CG12163). Note that *unc-119*, a homolog of the *C. elegans unc-119* gene [57], and *brinker* are within the intron of a larger gene, *CG1677*. It is not clear at this point which of these genes may be relevant to sleep. We also tested a mutation in *Vmat*, which transports dopamine, serotonin, and octopamine [58], monoamines known to affect sleep in *Drosophila*[38,59,60]. We saw significant differences in the expected sleep phenotypes for RNAi constructs of *CG14545* and *unc-119*, and a *P*-element insertion in *Vmat*.

All of these mutations and RNAi constructs exhibited significant effects in sleep phenotypes other than those tested, revealing pervasive pleiotropy. The BG01047 *P*-element insertion in *fz*, for example, was the most highly pleiotropic mutant we observed. We chose it to test it based on the night sleep *CV*_*E*_ and waking activity *CV*_*E*_ GWAS results, yet significant pleiotropic effects were observed in day and night bout number, day and night sleep, day and night average bout length, day and night bout number *CV*_*E*_, and day and night average bout length *CV*_*E*_ (Additional file 17). We observed 71 instances of pleiotropy in the tested candidate genes. Most of the unexpected pleiotropic effects (57 out of 71) occurred between sleep traits with statistically significant (although not necessarily high) genetic correlations in the DGRP population (Additional file 1). Pleiotropic effects of a mutation or RNAi allele that were not observed in the DGRP population can occur because genetic correlations in a natural population are indicative of pleiotropic effects of naturally occurring alleles that are in the same direction. Further, the effect sizes for most SNPs associated with sleep traits are in the range of 0.3 to 0.7 standard deviations, which is relatively small compared to the effects that might be expected with a null mutation. Thus, only when a mutation or RNAi construct is sufficiently severe do we observe the effect on a correlated sleep trait. Either or both of these factors could account for the unexpected pleiotropic effects of tested candidate genes on day bout number and waking activity, which were not correlated with the traits being tested (Additional file 17). We observed substantial pleiotropy at the level of individual SNPs as well as at the level of genes (Additional files 9 and 10). Given the generally rapid decay of LD in this population (over 10–30 bp) [20], non-overlapping SNPs that are associated with different traits may evolve independently, as has been observed previously [61]. In total, we observed 103 SNPs and 254 genes associated with two or more sleep traits.

The above discussion does not include SNPs associated with sleep traits in chromosomal inversions and regions of local LD. Three chromosomal inversions were significantly associated with sleep: *ln_2L_t*, which was significantly associated with day sleep, day sleep *CV*_*E*_, day bout number *CV*_*E*_, and night bout number *CV*_*E*_; *ln_2R_NS*, which was significantly associated with day average bout length mean and *CV*_*E*_ and day bout number; and *ln_3R_Mo*, which was significantly associated with day average bout length mean and *CV*_*E*_ and waking activity *CV*_*E*_. Furthermore, we found 193 and 418 regions of local LD for mean and *CV*_*E*_ sleep traits, respectively (Additional files 9 and 10). The vast majority of these LD regions (96.4%) contain 10 or fewer SNPs. We cannot distinguish which of the SNPs in these regions are potentially causal from the GWA analyses. It may be possible to determine the SNPs that are causal in LD regions by crossing lines of the DGRP together and performing a second association study once recombination has occurred over several generations. The chromosomal inversions present a more challenging problem, however, as recombination is effectively suppressed in these regions.

Here we associated fourteen sleep phenotypes with 2,490,195 SNPs in the DGRP. Although this analysis was blind to SNPs with minor allele frequencies less than 0.024 and non-SNP variants (insertions, deletions, translocations, transposable elements, copy number variants), we nevertheless found that a few SNPs in purely additive multi-SNP models could explain a large fraction of the genetic variance. This is in sharp contrast to the situation in human GWA studies [62,63], in which individuals SNPs explain only a small proportion of the total phenotypic variance. This is likely in part because sleep measures in this study were replicated for each genotype, which increases the statistical confidence in these measures. In addition, all flies were reared under controlled environmental conditions from staging the parental cultures until sleep and activity monitoring were completed at the adult stage. One potential reason for the ‘missing’ heritability in human association studies [62,63] is that the true causal variants are not common, and thus poorly tagged by common SNPs used in the genotyping platforms [64]. Our association study included all variants with frequencies greater than 2.4%, and found that the lower frequency variants had the largest effects, supporting the rare variants hypothesis.

## Conclusions

While much work remains to be done in order to understand the purpose of sleep, this study gives us some insight as to how genetic variation for sleep might be maintained. A number of genes for sleep have been previously identified by random mutagenesis screens in *Drosophila*. These studies have revealed genes important to sleep. However, these genes may be invariant in natural populations if, for instance, they are under strong purifying selection; if this is the case, they will not contribute to the maintenance of genetic variation in sleep. This study found that 19 of the genes already known to affect sleep in *Drosophila* affect genetic variation in a natural population as well (Additional file 13). The Gene Ontology analysis suggests that genes impacting sleep fall into very broad categories such as nervous system development and signal transduction. Further, genes in the *Egfr* signaling pathway were over-represented in this study, suggesting that polymorphisms in their component genes impact genetic variation in sleep. The *Egfr* pathway is a primary signaling cascade with multiple effects in the regulation of cell fate determination and morphogenesis and affects many downstream biological processes [65], implying that sleep is connected to many biological processes. The network derived from the SNP associations presents a working hypothesis for how polymorphic variants for sleep might interact. Note that many of the SNPs we identified herein are found in novel, computationally predicted genes having functions hypothesized on the basis of homology to genes in other species. A GO analysis using only the 579 computationally predicted genes from this study reveals additional pathways important for sleep. For example, most of the predicted genes are integral to the membrane, and many are predicted to catalyze the transport of substances from one side of the membrane to the other or to have a function in proteolysis.

A major challenge in human GWA studies is the inability to directly demonstrate that a candidate SNP causes the phenotypic variation. *Drosophila*, however, can be manipulated with transgenic approaches, crosses between different genotypes, and artificial selection procedures. This ability will enable us to determine whether the SNPs we identified herein are causal, whether the same SNPs have a role in sleep in different populations of *Drosophila*, whether epistatic interactions occur between SNPs, and the nature of the selective forces acting on sleep. Finally, this work forms the basis of a systems genetics analysis that will link polymorphic, molecular, and phenotypic variation for sleep in multiple environments.

## Methods

### Quantitative sleep phenotypes

We used the *Drosophila* Genetic Reference Panel (DGRP) [20] to assay sleep phenotypes. The DGRP was created by 20 generations of full sib mating of progeny of wild-caught, gravid females from Raleigh, North Carolina. We randomly divided the DGRP lines into four equal blocks. Flies were maintained under standard culture (cornmeal-molasses-agar medium, 25°C, 60-75% relative humidity) and lighting conditions (12-hour light: dark cycle). Sleep measurements were replicated four times for each block of lines. Eight flies of each sex were measured in each replicate, resulting in sleep measurements for 32 flies per sex per line. Eight male and eight female *w*^1118^; *Canton-S B* flies were measured in each replicate as a control. Virgin males and females were collected from each line and retained at 30 flies per same-sex vial to mitigate the effects of both social exposure [66] and mating [67] on sleep. We recorded seven continuous days of sleep and activity using the *Drosophila* Activity Monitoring System (Trikinetics, Waltham, MA), which measures the number of times a given fly crosses an infrared beam. Each fly was visually inspected at the end of the recording period; data from flies that did not live through the recording period were not used in the sleep calculations. A C^#^ program (R. Sean Barnes, personal communication) was used to calculate sleep duration, sleep bout number, average sleep bout length, and waking activity from the raw activity data. Sleep duration was calculated as any period of inactivity lasting five minutes or longer, as previously defined [11,12,22]. Waking activity was calculated as the number of times the fly crossed the infrared beam divided by the total time spent awake. Different genes influence sleep patterns during the day and night [7]; we therefore calculated sleep parameters separately for day and night. To assess the degree of sensitivity of sleep to the environment, we calculated the environmental coefficient of variation *CV*_*E*_ of each sleep parameter per line/sex/replicate as (*σ*_*E*_/μ) × 100 [33], where *σ*_*E*_ is the within-replicate environmental standard deviation and μ is the sleep trait averaged over each line, sex, and replicate.

### *Wolbachia pipientis* effects

The *Wolbachia* infection status for each DGRP line has been determined [20]. We assessed the degree to which sleep phenotypes were influenced by *Wolbachia* infection using the mixed-model ANOVA *Y* = *μ* + *I* + *S* + *I* × *S* + *L*(*I*) + *R*(*I* × *S* × *L*) + *ϵ*, where *I* designates infection status (fixed), *S* is sex (fixed), *L* is the DGRP line (random), *R* is the replicate effect (random), and *ϵ* is the error variance. We also used reduced models to examine the effect of infection status on sleep for each sex separately [20]. *Wolbachia* infection status significantly affected only one trait, day average bout length, and the effect was only present in males. Thus, we corrected male day average bout length line means to account for the influence of infection.

### Quantitative genetic analyses

We partitioned the variance in each sleep parameter and its respective environmental coefficient of variation *CV*_*E*_ using the ANOVA model: *Y* = *μ* + *B* + *S* + *L*(*B*) + *S* × *L*(*B*) + *R*(*B*) + *S* × *R*(*B*) + *R* × *L*(*B*) + *S* × *R* × *L*(*B*) + *ϵ*, where *L* (line), *B* (block) and *R* (replicate) are random effects, *S* (sex) is a fixed effect, and *ϵ* is the error variance. We used reduced models to partition the variance for each sex separately. Significant block effects (*P* < 0.05) were present for some sleep traits (Additional file 1). Significant block effects may be due to differences in the environment, or they may simply be the result of random sampling. To distinguish between these two possibilities, we analyzed sleep in the *w*^1118^; *Canton-S B* control line using the reduced model *Y* = *μ* + *B* + *S* + *B* × *S* + *R*(*B*) + *S* × *R*(*B*) + *ϵ*. None of the block terms were significant, suggesting that the significant differences seen between blocks in the raw sleep data are due to differences in the lines sampled, not environmental differences between blocks. We therefore used the raw sleep data in subsequent analyses.

We estimated variance components using the restricted maximum likelihood (REML) method. We calculated broad sense heritability as *H*^*2*^ = (*σ*^*2*^_*L*_ + *σ*^*2*^_*SL*_)/(*σ*^*2*^_*L*_ + *σ*^*2*^_*SL*_ + *σ*^*2*^_*E*_) for sexes combined, where *σ*^*2*^_*L*_ is the variance component among lines, *σ*^*2*^_*SL*_ is the line-by-sex variance component, and *σ*^*2*^_*E*_ is the sum of all other sources of variation. We used *H*^*2*^ = *σ*^*2*^_*L*_/(*σ*^*2*^_*L*_ + *σ*^*2*^_*E*_) to estimate broad-sense heritability for each sex separately. We calculated the cross-sex genetic correlation *r*_*MF*_ as *σ*^*2*^_*L*_/√(*σ*^*2*^_*LM*_ × *σ*^*2*^_*LF*_), where *σ*^*2*^_*L*_ is the variance component among lines for sexes combined, *σ*^*2*^_*LM*_ is the variance component among lines for males, and *σ*^*2*^_*LF*_ is the variance component among lines for females. We calculated the genetic correlation *r*_*G*_ between each sleep trait *r*_*G*_ = *cov*_12_/√(*σ*_*L1*_^*2*^ × *σ*_*L2*_^*2*^) [27], where *cov*_12_ is the covariance between traits 1 and 2, and *σ*_*L1*_^*2*^ and *σ*_*L2*_^*2*^ are the among-line variances for traits 1 and 2, respectively.

### Genotype-phenotype associations

We associated the line mean of each sleep parameter with all segregating sites in the DGRP present in four or more DGRP lines, and having sequence coverage levels greater than two and less than thirty [20]. We used the ANOVA model *Y* = *μ* + *SNP* + *S* + *SNP* × *S* + *L*(*SNP*) + *ϵ* to evaluate each segregating site [20]. *SNP* is the genotype effect, while *L* and *S* are as defined above. Genotype-phenotype associations were also performed for males and females separately using the reduced model *Y* = *μ* + *SNP* + *ϵ*. We defined our threshold *P*-values for each sleep trait separately by calculating the false discovery rate (FDR) using the standard Benjamini-Hochberg procedure [68]. Significant SNPs were designated as those with FDR ≤ 0.01. We calculated the standardized effect size (*a*/*σ*_*P*_) as one-half the difference between marker classes divided by the overall phenotypic standard deviation [27] and the linkage disequilibrium among significant markers using *r*^2^[69]. It is not likely that all 2,490,195 SNPs in the DGRP act independently of one another. The genetic variance explained by each SNP can be overestimated if population structure is present in the DGRP, if the SNPs are in linkage disequilibrium, or if the SNPs are correlated with one another by chance due to the small size of the DGRP. We saw evidence of a deviation from the expected *P*-value distribution in the original analysis. We therefore checked for population structure by incorporating the DGRP relationship matrix into the genome-wide association model using Fast-LMM [70,71]. Incorporating the relationship matrix did not explain the deviation of the observed *P*-value distribution from the expected distribution, suggesting that there was no population structure due to population admixture in the DGRP. We associated the presence or absence of chromosomal inversions present in the DGRP (M. A. Carbone, A. Yamamoto, Y. Inoue, and T. F. C. Mackay, personal communication) with sleep phenotypes using the model *Y* = *μ* + *Inv* + *S* + *Inv* × *S* + ϵ, where *Inv* denotes the presence or absence of the inversion, and *S* and ϵ are defined above. As these chromosomal inversions and LD blocks are the likely source of the deviations of the observed distribution of *P*-values from expectation under the null hypothesis of no association, we counted significant SNPs within these regions as a single SNP. We also performed iterative GWA analyses that incorporated the additive effects of multiple SNPs. We used the model, *Y* = μ + *SNP*_1_ + *SNP*_2_ + *SNP*_3_ + … *SNP*_N_ + ϵ, where *SNP*_1_, *SNP*_2_, *SNP*_3_, …, *SNP*_N_ are the most significant SNPs fitted in succession into the model. In this model, *SNP*_1_ is the most highly significant SNP in the original genome-wide analysis. *SNP*_2_ is the most highly significant SNP in the genome-wide analysis after *SNP*_1_ is fitted into the model, *SNP*_3_ is the most highly significant SNP in the genome-wide analysis after *SNP*_1_ and *SNP*_2_ are fitted into the model, and so on. We continued to fit SNPs to the model until the *r*^2^ parameter was maximal.

### Verification of genotype-phenotype associations

We used the SNP locations to determine which SNPs were located within annotated *D. melanogaster* genes for all mean and *CV*_*E*_ sleep traits (Additional files 9 and 10). We searched the DAVID bioinformatics database [30] for pathways with significant enrichment for these genes. The *Epidermal growth factor receptor* (*Egfr*) pathway was highly significant. We then used BIOGRID [32], a data base compiled from the literature of known protein-protein and genetic interactions, to search our gene list for interactions with the genes in the *Egfr* pathway. We identified candidate genes previously reported to interact with this pathway, and constructed a network around the *Egfr* pathway based on these interactions. We chose mutations in highly-connected genes from this pathway for further testing. We used homozygous *P*-element insertion lines from the Exelixis and MB stock collections, which were produced in an isogenic background and therefore have an isogenic control [72,73]. We tested *Minos* mutations for *Vmat* (*Mi*{*ET1*}*Vmat*^MB04557^) and *tkv* (*Mi*{*ET1*}*tkv*^MB02285^. We tested the Exelixis mutation *Pbac*{*WH*}*Ubx*^f07161^ in *Ultrabithorax*. We also tested BG01047, a line with a *P*[GT1] *P*-element insertion in the gene *fz*[74]. This insertion also has an isogenic background, *w*^1118^; *Canton-S*. In addition, we used RNA interference to knock down the expression of selected genes in all fly neurons. We tested the effects of RNAi knockdown in the following genes and compared them to the isogenic control line *y*,*w*^1118^;*P*{attP, *y*^+^, *w*^3^}: *brinker*^101887^, *CG11163*^107931^, *CG12163*^107589^, *CG14545*^103660^, *Hey*^103570^, *scribbled*^100363^, and *unc-119*^107987^ (Vienna *Drosophila* RNAi Center) [75]. We used the *P*{[*w*^+^Mc] = GAL4-*elav*.L}2/*CyO* line (8765) from the Bloomington Stock Center to drive expression pan-neuronally.

### Ethics statement

The research performed in this study on the fruit fly, *Drosophila melanogaster*, did not require approval by an ethics committee.

## Competing interests

The authors declare that they have no competing interests.

## Authors’ contributions

STH and TFCM conceived of the experiment and wrote the paper. STH measured sleep in the DGRP, tested the mutant lines and analyzed the data. LJM tested the RNAi lines. All authors read and approved the final manuscript.

## Supplementary Material

Additional file 1 **Quantitative genetic analyses of variance of mean and *CV***_***E ***_**sleep phenotypes in the DGRP lines.** Click here for file

Additional file 2 **Histograms of mean and coefficient of environmental variation (*CV***_***E***_**) for bout number, average bout length, and waking activity.** Click here for file

Additional file 3 Mean phenotypic values of sleep quantitative traits. Click here for file

Additional file 4 Distribution of significant SNPs by site class. Click here for file

Additional file 5 Q-Q plots for males. Click here for file

Additional file 6 Q-Q plots for females. Click here for file

Additional file 7 ***P*-value histograms for males.** Click here for file

Additional file 8 ***P*-value histograms for females.** Click here for file

Additional file 9 GWA results for mean sleep traits. Click here for file

Additional file 10 **GWA results for sleep trait*CV***_***E***_**s.** Click here for file

Additional file 11 Minor allele frequency versus normalized effect size. Click here for file

Additional file 12 Additive multiple SNP predictive model results. Click here for file

Additional file 13 Summary characteristics of genes implicated in the GWAS. Click here for file

Additional file 14 Gene Ontology (GO) categories. Click here for file

Additional file 15 **Genes interacting with *Epidermal growth factor receptor (Egfr*) pathway.** Click here for file

Additional file 16 Mutant and RNAi verification test results. Click here for file

Additional file 17 Pleiotropic effects in sleep candidate genes. Click here for file
