# Supplementary material for: Genome-wide association study of sleep in Drosophila melanogaster
Source: BMC Genomics. 2013 Apr 25;14:281. doi: 10.1186/1471-2164-14-281 (PMC3644253; doi:10.1186/1471-2164-14-281)
Supplement: Additional file 1 — Quantitative genetic analyses of variance of mean and CVE sleep phenotypes in the DGRP lines. [file 1471-2164-14-281-S1.pdf]

Additional file 1. Quantitative genetic analysis of sleep phenotypes in the DGRP lines.

Analyses of variance of mean sleep phenotypes.

d.f., degrees of freedom; MS, Type III Mean Squares; F, F ratio statistic; *P*, *P*-value for F ratio statistic; σ^2, variance component; H^2, broad sense heritability; r_{MF}, cross-sex genetic correlation; *, MS and σ^2 multiplied by 10^3.

Phenotype	Analysis	Source	*d.f.*	MS	F	*P*	σ^2	H^2	r_{MF}
Night sleep (min.)	Combined Sex	Block	3	1053739.68	1.96	0.1234	206.43	0.54	0.76
		Sex	1	763.09	0.01	0.9190	Fixed		
		Line(Block)	164	441081.10	5.99	<0.0001	6202.78		
		Sex×Line(Block)	164	71205.22	8.04	<0.0001	2066.41		
		Rep(Block)	12	110791.73	7.89	0.0001	151.17		
		Sex×Rep(Block)	12	11634.82	1.31	0.2065	7.49		
		Rep×Line(Block)	492	11282.03	1.27	0.0038	155.54		
		Sex×Rep×Line(Block)	490	8866.51	1.44	<0.0001	349.39		
		Error	8897	6167.76	--	--	6172.65		
	Males	Block	3	323954.85	1.28	0.2832	56.14	0.56	
		Line(Block)	164	225069.41	26.28	<0.0001	7198.23		
		Rep(Block)	12	37959.11	4.43	<0.0001	91.50		
		Line×Rep(Block)	490	8580.19	1.68	<0.0001	454.40		
		Error	4462	5099.14	--	--	5099.57		
	Females	Block	3	788080.04	2.19	0.0926	342.63	0.53	
		Line(Block)	164	288060.57	25.11	<0.0001	9345.94		
		Rep(Block)	12	84748.26	7.39	<0.0001	227.06		
		Line×Rep(Block)	492	11489.73	1.59	<0.0001	549.57		
		Error	4435	7242.89	--	--	7254.30		
Day sleep (min.)	Combined Sex	Block	3	1706424.93	2.52	0.0618	416.49	0.49	0.70
		Sex	1	35593659.52	280.04	<0.0001	Fixed		
		Line(Block)	164	518986.18	4.78	<0.0001	6693.28		
		Sex×Line(Block)	164	105107.94	6.30	<0.0001	2985.18		
		Rep(Block)	12	180974.81	4.20	0.0063	202.83		
		Sex×Rep(Block)	12	39568.82	2.37	0.0056	80.60		
		Rep×Line(Block)	492	20231.49	1.21	0.0171	231.69		
		Sex×Rep×Line(Block)	490	16713.17	2.05	<0.0001	1123.85		
		Error	8897	8146.71	--	--	8149.32		
	Males	Block	3	1157289.69	2.91	0.0414	590.02	0.46	
		Line(Block)	164	261022.90	14.19	<0.0001	7916.75		
		Rep(Block)	12	157967.48	8.59	<0.0001	423.73		
		Line×Rep(Block)	490	18443.77	2.71	<0.0001	1521.89		
		Error	4462	6805.40	--	--	6806.32		
	Females	Block	3	768835.63	1.88	0.1357	294.00	0.51	
		Line(Block)	164	364092.68	19.74	<0.0001	11425.29		
		Rep(Block)	12	65570.78	3.56	<0.0001	140.79		
		Line×Rep(Block)	492	18477.80	1.95	<0.0001	1180.41		
		Error	4435	9496.18	--	--	9502.57		
Night bout number	Combined Sex	Block	3	3480.80	2.47	0.0679	0.85	0.42	0.74
		Sex	1	2609.38	16.37	0.0001	Fixed		
		Line(Block)	164	968.57	5.41	<0.0001	13.08		
		Sex×Line(Block)	164	178.04	4.87	<0.0001	4.71		
		Rep(Block)	12	484.70	24.51	<0.0001	0.70		
		Sex×Rep(Block)	12	18.77	0.51	0.9062	0.00		
		Rep*Line(Block)	492	37.61	1.03	0.3783	0.07		
		Sex×Rep×Line(Block)	490	36.58	1.71	<0.0001	1.96		
		Error	8897	21.40	--	--	21.40		
	Males	Block	3	1017.78	1.40	0.2496	0.24	0.43	
		Line(Block)	164	525.38	14.94	<0.0001	16.16		
		Rep(Block)	12	242.37	6.90	<0.0001	0.63		
		Line×Rep(Block)	490	35.23	1.96	<0.0001	2.26		
		Error	4462	18.00	--	--	18.00		
	Females	Block	3	2681.41	3.15	0.0288	1.52	0.40	
		Line(Block)	164	626.56	16.12	<0.0001	19.36		
		Rep(Block)	12	266.89	6.87	<0.0001	0.71		
		Line×Rep(Block)	492	38.94	1.57	<0.0001	1.85		
		Error	4435	24.82	--	--	24.84		

Phenotype	Analysis	Source	*d.f.*	MS	F	*P*	σ^2	H^2	r_{MF}
Day bout number	Combined Sex	Block	3	630.83	0.42	0.7427	0.00	0.33	0.58
		Sex	1	7064.63	12.23	0.0014	Fixed		
		Line(Block)	164	952.74	3.01	<0.0001	10.48		
		Sex×Line(Block)	164	312.71	4.38	<0.0001	8.01		
		Rep(Block)	12	648.94	1.88	0.1423	0.39		
		Sex×Rep(Block)	12	341.11	4.78	<0.0001	0.89		
		Rep×Line(Block)	492	75.68	1.06	0.2646	0.29		
		Sex×Rep×Line(Block)	490	71.53	2.35	<0.0001	5.40		
		Error	8897	30.50	--	--	30.50		
	Males	Block	3	704.18	0.54	0.6565	0.00	0.29	
		Line(Block)	164	514.41	6.65	<0.0001	14.42		
		Rep(Block)	12	864.94	11.20	<0.0001	2.30		
		Line×Rep(Block)	490	77.52	3.04	<0.0001	6.82		
		Error	4462	25.51	--	--	25.52		
	Females	Block	3	645.04	0.80	0.4939	0.00	0.36	
		Line(Block)	164	752.84	10.76	<0.0001	22.64		
		Rep(Block)	12	123.74	1.77	0.0504	0.17		
		Line×Rep(Block)	492	70.13	1.97	<0.0001	4.57		
		Error	4435	35.51	--	--	35.50		
Night avg. bout length (min.)	Combined Sex	Block	3	1552880.11	4.37	0.0063	470.79	0.38	0.77
		Sex	1	45339.74	1.06	0.3068	Fixed		
		Line(Block)	164	263510.85	6.13	<0.0001	3653.06		
		Sex×Line(Block)	164	40009.34	4.27	<0.0001	1113.43		
		Rep(Block)	12	105937.84	6.85	0.0002	134.00		
		Sex×Rep(Block)	12	12490.92	1.33	0.1958	31.00		
		Rep×Line(Block)	492	12373.68	1.32	0.0011	186.86		
		Sex×Rep×Line(Block)	490	9380.87	1.41	<0.0001	353.35		
		Error	8896	6638.25	--	--	6648.70		
	Males	Block	3	254426.03	1.30	0.2794	41.55	0.41	
		Line(Block)	164	149561.92	14.35	<0.0001	4643.52		
		Rep(Block)	12	57817.56	5.55	<0.0001	146.57		
		Line×Rep(Block)	490	10439.10	1.73	<0.0001	575.91		
		Error	4462	6017.66	--	--	6021.76		
	Females	Block	3	1585366.18	7.69	0.0001	1097.66	0.35	
		Line(Block)	164	156048.96	13.93	<0.0001	4807.79		
		Rep(Block)	12	62233.33	5.56	<0.0001	174.13		
		Line×Rep(Block)	492	11219.66	1.54	<0.0001	502.77		
		Error	4434	7262.75	--	--	7281.11		
Day avg. bout length (min.)	Combined Sex	Block	3	45468.01	2.31	0.0904	10.19	0.19	0.69
		Sex	1	755971.49	63.70	<0.0001	Fixed		
		Line(Block)	164	11538.82	1.97	<0.0001	93.04		
		Sex×Line(Block)	164	5626.32	3.41	<0.0001	135.33		
		Rep(Block)	12	10139.65	1.23	0.3582	1.86		
		Sex×Rep(Block)	12	7990.32	4.84	<0.0001	21.98		
		Rep×Line(Block)	492	1892.61	1.15	0.0661	16.78		
		Sex×Rep×Line(Block)	490	1652.65	2.04	<0.0001	109.21		
		Error	8896	811.86	--	--	813.17		
	Males	Block	3	62982.80	2.13	0.1151	26.28	0.21	
		Line(Block)	164	15436.23	5.03	<0.0001	413.40		
		Rep(Block)	12	17374.11	5.66	<0.0001	43.66		
		Line×Rep(Block)	490	3078.90	2.45	<0.0001	238.53		
		Error	4462	1259.16	--	--	1260.82		
	Females	Block	3	3284.37	1.54	0.2141	0.82	0.10	
		Line(Block)	164	1760.60	3.72	<0.0001	43.39		
		Rep(Block)	12	855.19	1.81	0.0442	1.24		
		Line×Rep(Block)	492	473.67	1.31	<0.0001	12.08		
		Error	4434	361.72	--	--	363.85		

Phenotype	Analysis	Source	*d.f.*	MS	F	*P*	σ^2	H^2	r_{MF}
Waking activity (cts/min.)	Combined Sex	Block	3	2190.00	0.43	0.7346	0.00	0.39	0.84
		Sex	1	242031.00	247.27	<0.0001	Fixed		
		Line(Block)	164	4979.80	6.14	<0.0001	68.54		
		Sex×Line(Block)	164	798.20	4.35	<0.0001	20.62		
		Rep(Block)	12	372.10	0.97	0.5182	0.00		
		Sex×Rep(Block)	12	370.38	2.02	0.0209	0.53		
		Rep×Line(Block)	492	196.20	1.07	0.2281	0.80		
		Sex×Rep×Line(Block)	490	183.49	1.42	<0.0001	7.13		
		Error	8897	129.16	--	--	129.18		
	Males	Block	3	1715.10	0.41	0.7496	0.00	0.41	
		Line(Block)	164	3944.04	18.01	<0.0001	124.67		
		Rep(Block)	12	533.00	2.43	0.0044	0.94		
		Line×Rep(Block)	490	0.22	1.30	<0.0001	6.64		
		Error	4462	0.17	--	--	168.39		
	Females	Block	3	1.14	0.61	0.6088	0.00	0.35	
		Line(Block)	164	1.82	11.43	<0.0001	53.96		
		Rep(Block)	12	0.21	1.31	0.2094	0.12		
		Line×Rep(Block)	492	0.16	1.78	<0.0001	9.18		
		Error	4435	0.09	--	--	89.78		

Analyses of variance of sleep coefficients of environmental variation (CV_E) in the DGRP lines.

d.f., degrees of freedom; MS, Type III Mean Squares; F, F ratio statistic; *P*, *P*-value for F ratio statistic; σ^2, variance component; H^2, broad sense heritability; r_{MF}, cross-sex genetic correlation.

**Phenotype**	**Analysis**	**Source**	***d.f.***	**MS**	**F**	***P***	σ^2	H^2	r_{MF}
	Combined Sex	Block	3	394.19	0.54	0.6556	0.00	0.72	0.74
		Sex	1	2964.36	17.09	0.0001	--		
		Line(Block)	164	730.27	4.21	<0.0001	68.95		
		Sex×Line(Block)	167	173.46	4.35	<0.0001	33.45		
Night sleep		Error	1006	39.88	--	--	39.88		
CV_E	Males	Block	3	115.72	0.43	0.7349	0.00	0.69	
		Line(Block)	164	272.10	9.97	<0.0001	60.64		
		Error	502	27.28	--	--	27.28		
	Females	Block	3	391.49	0.62	0.6042	0.00	0.73	
		Line(Block)	164	633.34	12.08	<0.0001	144.14		
		Error	504	52.43	--	--	52.43		
	Combined Sex	Block	3	3031.18	3.53	0.0163	6.58	0.55	0.61
		Sex	1	68301.84	223.13	<0.0001	--		
		Line(Block)	164	859.84	2.81	<0.0001	69.22		
		Sex×Line(Block)	167	306.16	3.29	<0.0001	53.32		
Day sleep		Error	1006	93.19	--	--	93.18		
CV_E	Males	Block	3	573.55	1.55	0.2043	1.14	0.55	
		Line(Block)	164	370.97	5.99	<0.0001	77.46		
		Error	502	61.96			61.96		
	Females	Block	3	3593.88	4.61	0.0040	16.97	0.54	
		Line(Block)	164	779.74	6.27	<0.0001	163.87		
		Error	504	124.29	--	--	124.29		
	Combined Sex	Block	3	5144.01	8.15	<0.0001	13.59	0.31	0.71
		Sex	1	4380.57	18.84	<0.0001	--		
		Line(Block)	164	631.16	2.71	<0.0001	49.84		
		Sex×Line(Block)	167	232.50	1.60	<0.0001	21.80		
Night bout		Error	1006	144.96	--	--	145.02		
number	Males	Block	3	959.83	2.33	0.0759	3.32	0.33	
CV_E		Line(Block)	164	411.44	3.05	<0.0001	69.40		
		Error	502	134.98	--	--	135.02		
	Females	Block	3	5083.51	11.57	<0.0001	27.73	0.28	
		Line(Block)	164	439.45	2.84	<0.0001	71.13		
		Error	504	154.91	--	--	154.91		
	Combined Sex	Block	3	1101.03	2.11	0.1011	1.74	0.47	0.38
		Sex	1	3463.15	11.12	0.0011	--		
		Line(Block)	164	522.11	1.68	0.0005	26.32		
		Sex×Line(Block)	167	311.62	3.42	<0.0001	55.22		
Day bout		Error	1006	91.09	--	--	91.08		
number	Males	Block	3	510.69	2.15	0.0958	1.63	0.31	
CV_E		Line(Block)	164	237.56	2.79	<0.0001	38.20		
		Error	502	85.11	--	--	85.08		
	Females	Block	3	1093.30	1.84	0.1412	2.95	0.55	
		Line(Block)	164	592.92	6.11	<0.0001	124.05		
		Error	504	97.05	--	--	97.15		

Phenotype	Analysis	Source	*d.f.*	MS	F	*P*	σ^2	H^2	r_{MF}
Night avg. bout length CV_E	Combined Sex	Block	3	10057.95	7.89	0.0001	26.13	0.15	0.76
		Sex	1	10369.93	15.71	0.0001	--		
		Line(Block)	164	1275.06	1.93	<0.0001	76.98		
		Sex×Line(Block)	167	660.06	1.17	0.0812	23.91		
		Error	1006	563.12	--	--	563.31		
	Males	Block	3	3473.51	3.48	0.0174	14.45	0.14	
		Line(Block)	164	999.49	1.66	<0.0001	99.67		
		Error	502	600.71	--	--	601.07		
	Females	Block	3	6904.37	7.34	0.0001	35.77	0.16	
		Line(Block)	164	940.16	1.79	<0.0001	103.62		
		Error	504	525.68	--	--	525.68		
Day avg. bout length CV_E	Combined Sex	Block	3	3073.13	2.73	0.0458	5.76	0.06	0.96
		Sex	1	16643.68	21.81	<0.0001	--		
		Line(Block)	164	1126.65	1.48	0.0063	45.44		
		Sex×Line(Block)	167	763.12	1.02	0.4138	4.53		
		Error	1006	746.25	--	--	745.97		
	Males	Block	3	2088.19	2.00	0.1162	6.34	0.05	
		Line(Block)	164	1044.82	1.22	0.0515	48.28		
		Error	502	854.12	--	--	853.51		
	Females	Block	3	2817.78	3.42	0.0188	12.15	0.07	
		Line(Block)	164	824.97	1.29	0.0190	46.58		
		Error	504	638.80	--	--	638.80		
Waking activity CV_E	Combined Sex	Block	3	137.89	0.58	0.6278	0.00	0.24	0.98
		Sex	1	35.75	0.47	0.4924	--		
		Line(Block)	164	237.07	3.14	<0.0001	20.27		
		Sex×Line(Block)	167	75.51	1.11	0.1823	1.80		
		Error	1006	68.15	--	--	68.19		
	Males	Block	3	202.00	1.08	0.3607	0.10	0.3	
		Line(Block)	164	187.76	2.74	<0.0001	29.85		
		Error	502	68.61	--	--	68.59		
	Females	Block	3	88.26	0.70	0.5509	0.00	0.17	
		Line(Block)	164	125.36	1.85	<0.0001	14.25		
		Error	504	67.68	--	--	67.68		

Phenotypic and genetic correlations. Phenotypic correlations are above the diagonal; genetic correlations are below the diagonal. Bold indicates that the correlation is significantly different from zero.

Trait	Night sleep	Day sleep	Night bout number	Day bout number	Night avg. bout length	Day avg. bout length	Waking activity	Night sleep CV_E	Day sleep CV_E	Night bout number CV_E	Day bout number CV_E	Night avg. bout length CV_E	Day avg. bout length CV_E	Waking activity CV_E
Night sleep		**0.20**	**-0.29**	-0.02	**0.31**	0.10	0.02	**-0.52**	**-0.18**	**0.23**	-0.06	0.00	0.04	0.08
Day sleep	**0.38**		-0.11	0.10	0.11	**0.17**	0.05	**-0.21**	**-0.41**	0.03	**-0.19**	-0.06	0.05	0.09
Night bout number	**-0.61**	**-0.25**		0.11	**-0.33**	-0.10	-0.05	**0.26**	0.08	**-0.27**	-0.04	0.03	-0.06	-0.06
Day bout number	-0.05	**0.24**	**0.31**		-0.10	-0.08	-0.15	0.02	-0.11	-0.07	**-0.20**	0.02	-0.05	0.01
Night avg. bout length	**0.69**	**0.26**	**-0.82**	**-0.28**		0.10	0.06	**-0.28**	-0.08	**0.28**	0.03	-0.02	0.06	0.05
Day avg. bout length	**0.29**	**0.54**	**-0.36**	**-0.34**	**0.39**		0.12	-0.09	-0.13	0.06	0.03	-0.01	0.09	0.07
Waking activity	0.05	0.11	-0.13	**-0.42**	0.15	**0.44**		-0.04	-0.03	0.00	0.10	-0.03	0.04	0.10
Night sleep CV_E	**-0.83**	**-0.36**	**0.47**	0.04	**-0.55**	**-0.25**	-0.07		**0.24**	-0.14	0.10	0.08	-0.01	-0.06
Day sleep CV_E	**-0.33**	**-0.78**	**0.18**	**-0.27**	**-0.17**	**-0.39**	-0.07	**0.39**		0.02	**0.31**	0.10	0.02	-0.02
Night bout number CV_E	**0.56**	0.08	**-0.75**	**-0.23**	**0.80**	**0.25**	0.00	**-0.30**	0.05		0.08	0.11	0.07	0.02
Day bout number CV_E	-0.11	**-0.41**	-0.10	**-0.51**	0.08	0.11	0.22	**0.18**	**0.61**	**0.20**		0.04	0.09	0.06
Night avg. bout length CV_E	-0.01	**-0.22**	0.10	0.10	-0.08	**0.39**	-0.13	**0.25**	**0.36**	**0.53**	**0.16**		0.05	0.01
Day avg. bout length CV_E	**0.24**	**0.28**	**-0.35**	**-0.37**	**0.38**	**0.86**	**0.26**	-0.06	0.10	**0.52**	**0.50**	**0.57**		0.07
Waking activity CV_E	**0.22**	**0.27**	**-0.17**	0.02	**0.18**	**0.33**	**0.33**	-0.13	-0.06	0.06	**0.17**	0.05	**0.55**	
