# Supplementary material for: Genome-wide association study of sleep in Drosophila melanogaster
Source: BMC Genomics. 2013 Apr 25;14:281. doi: 10.1186/1471-2164-14-281 (PMC3644253; doi:10.1186/1471-2164-14-281)
Supplement: Additional file 2 — Histograms of mean and coefficient of environmental variation (CVE) for bout number, average bout length, and waking activity. [file 1471-2164-14-281-S2.pdf]

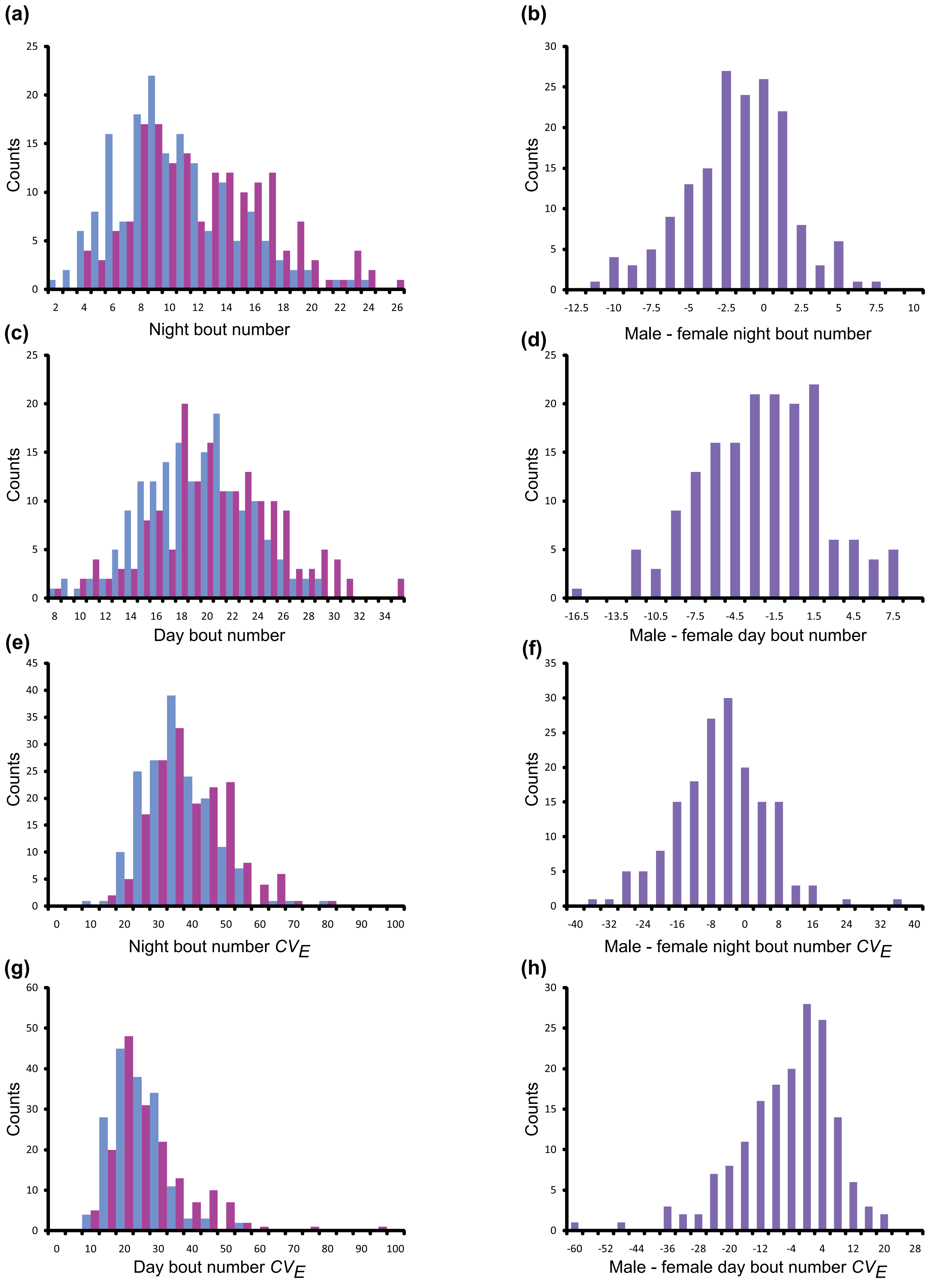
(a)
Counts
Night bout number
(b)
Counts
Male - female night bout number
(c)
Counts
Day bout number
(d)
Counts
Male - female day bout number
(e)
Counts
Night bout number CV_E
(f)
Counts
Male - female night bout number CV_E
(g)
Counts
Day bout number CV_E
(h)
Counts
Male - female day bout number CV_E

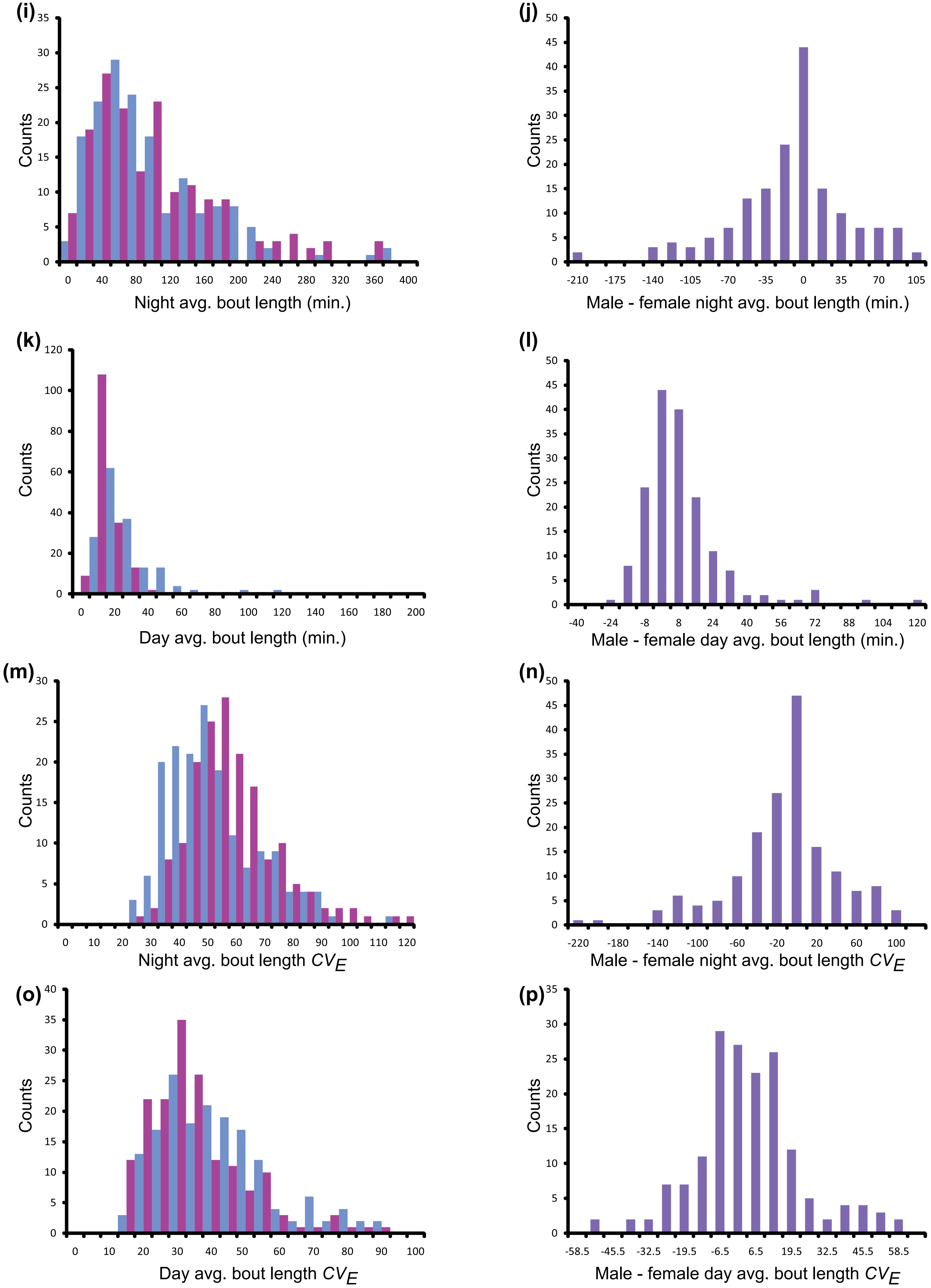
(i)
Counts
0 40 80 120 160 200 240 280 320 360 400
Night avg. bout length (min.)
(j)
Counts
-210 -175 -140 -105 -70 -35 0 35 70 105
Male - female night avg. bout length (min.)
(k)
Counts
0 20 40 60 80 100 120 140 160 180 200
Day avg. bout length (min.)
(l)
Counts
-40 -24 -8 8 24 40 56 72 88 104 120
Male - female day avg. bout length (min.)
(m)
Counts
0 10 20 30 40 50 60 70 80 90 100 110 120
Night avg. bout length CV_E
(n)
Counts
-220 -180 -140 -100 -60 -20 20 60 100
Male - female night avg. bout length CV_E
(o)
Counts
0 10 20 30 40 50 60 70 80 90 100
Day avg. bout length CV_E
(p)
Counts
-58.5 -45.5 -32.5 -19.5 -6.5 6.5 19.5 32.5 45.5 58.5
Male - female day avg. bout length CV_E

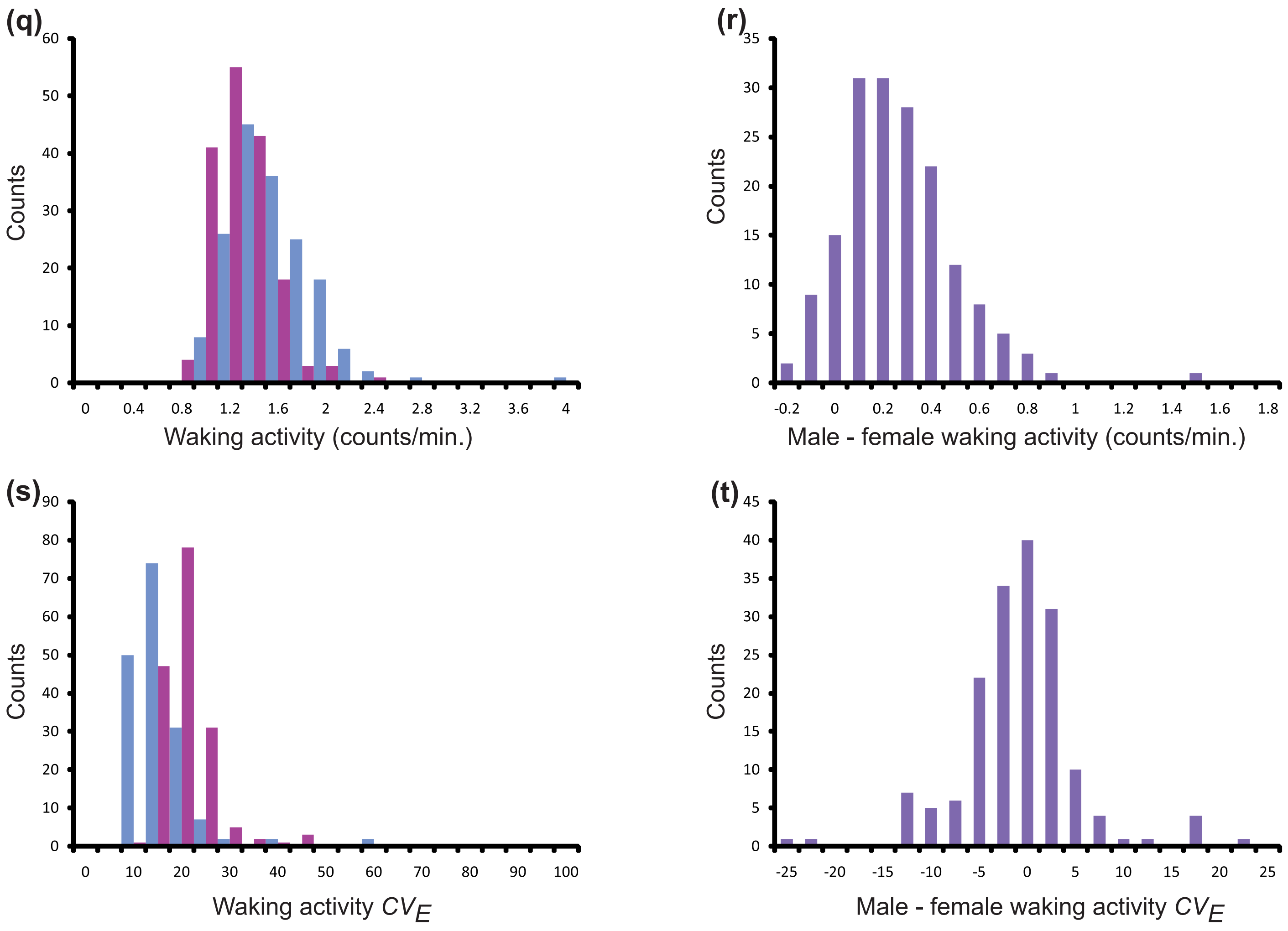

Additional file 2. Histograms of mean and coefficient of environmental variation (CV_E).
Male lines are shown in blue, while female lines are shown in pink. The difference in line means (male - female) is shown by purple bars. (a) Night bout number. (b) Male - female night bout number. (c) Day bout number. (d) Male - female day bout number. (e) Night bout number CV_E. (f) Male - female night bout number CV_E. (g) Day bout number CV_E. (h) Male - female day bout number CV_E. (i) Night avg. bout length. (j) Male - female night avg. bout length. (k) Day avg. bout length. (l) Male - female day avg. bout length. (m) Night avg. bout length CV_E. (n) Male - female night avg. bout length CV_E. (o) Day avg. bout length CV_E. (p) Male - female day avg. bout length CV_E. (q) waking activity. (r) Male - female waking activity. (s) Waking activity CV_E. (t) Male - female waking activity CV_E.
