# Supplementary figures and images for: Genome-wide association study of sleep in Drosophila melanogaster
Source: BMC Genomics. 2013 Apr 25;14:281. doi: 10.1186/1471-2164-14-281 (PMC3644253; doi:10.1186/1471-2164-14-281)

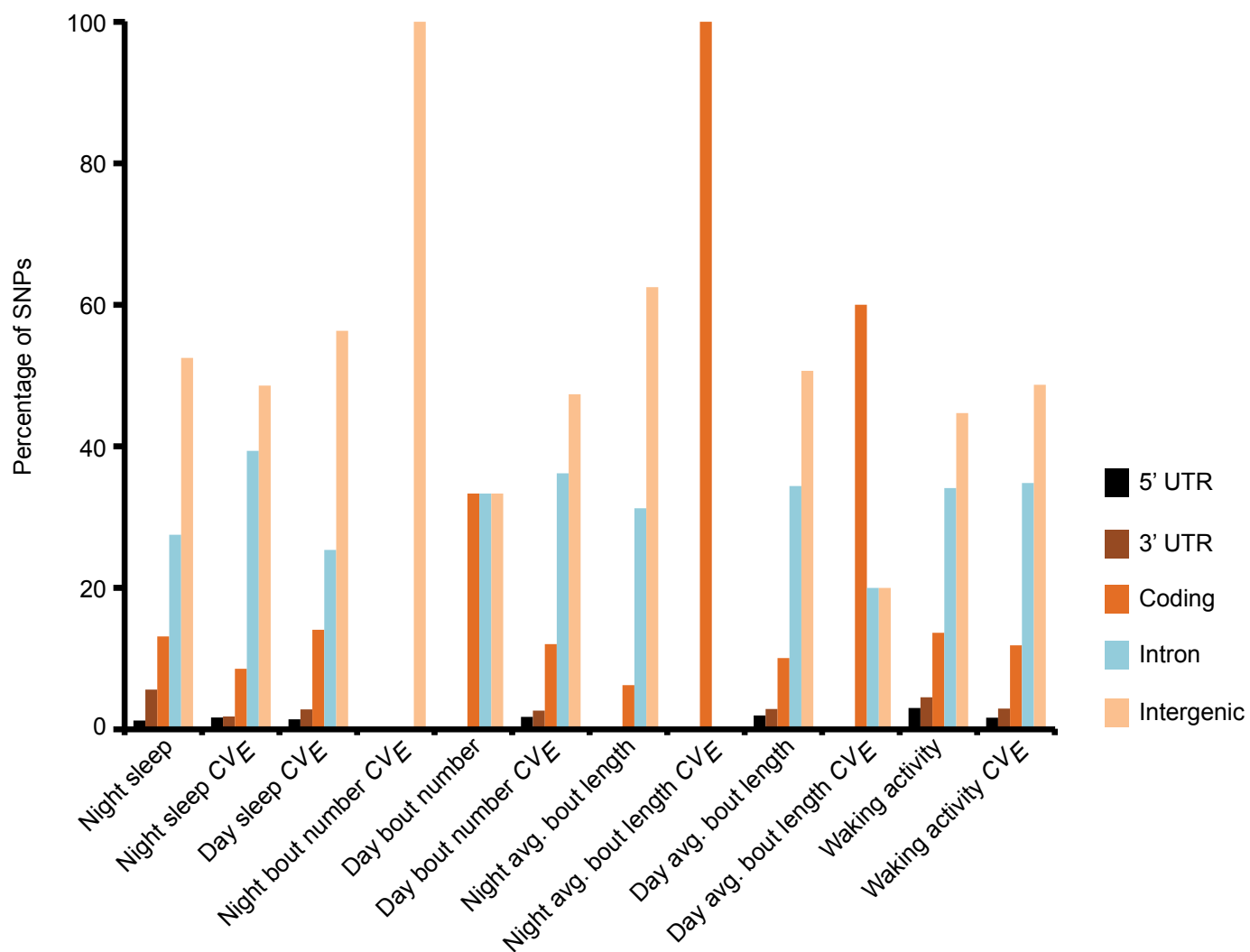

Additional file 4. Distribution of significant SNPs by site class.

Supplement: Additional file 4 — Distribution of significant SNPs by site class. [file 1471-2164-14-281-S4.pdf]
