# Supplementary material for: Genome-wide association study of sleep in Drosophila melanogaster
Source: BMC Genomics. 2013 Apr 25;14:281. doi: 10.1186/1471-2164-14-281 (PMC3644253; doi:10.1186/1471-2164-14-281)
Supplement: Additional file 5 — Q-Q plots for males. [file 1471-2164-14-281-S5.pdf]

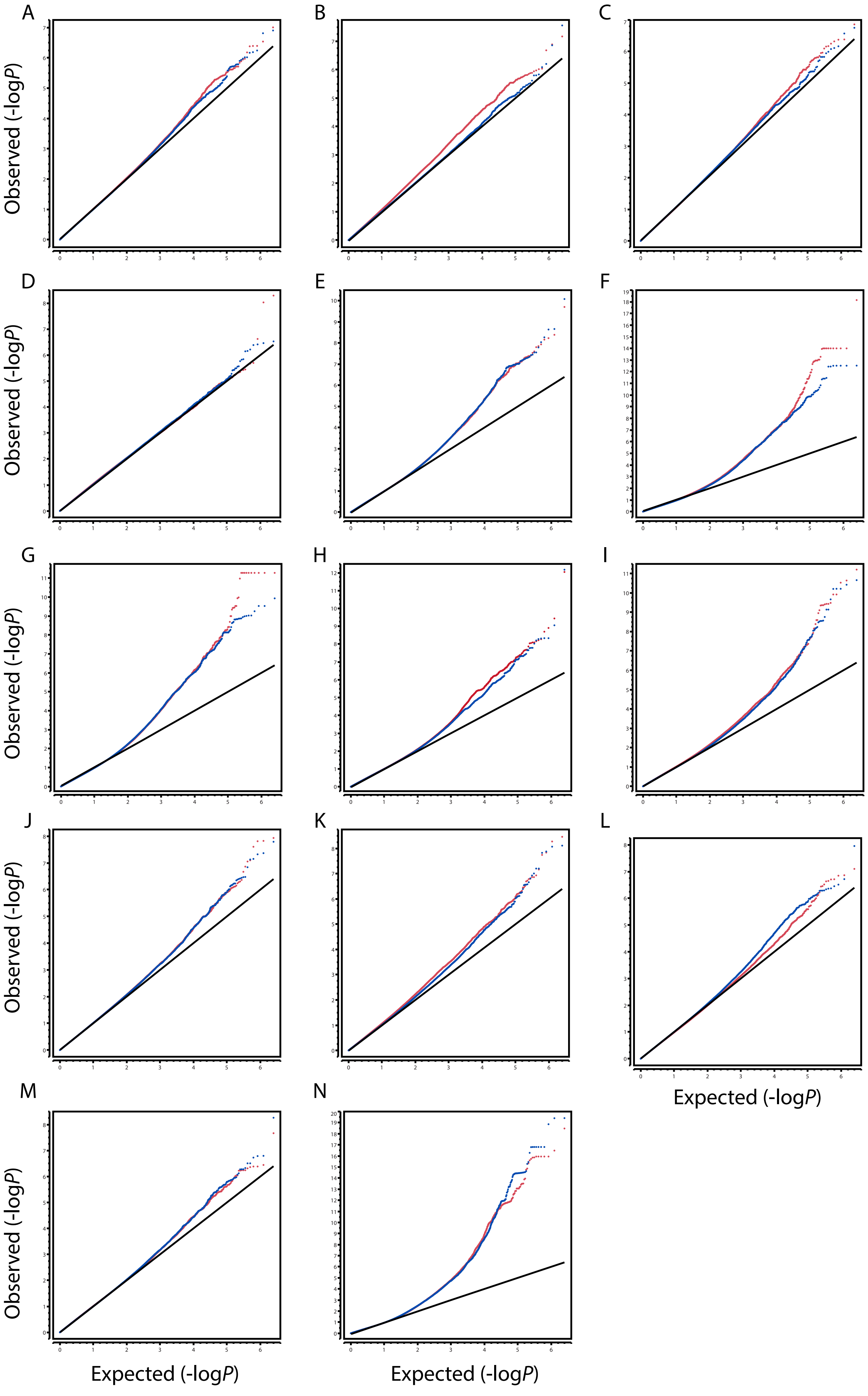
A
B
C
D
E
F
G
H
I
J
K
L
M
N
Observed (-logP)
Expected (-logP)

Additional file 5. Q-Q plots for males.

The black line indicates the expected *P*-value distribution. Red circles show the distribution of the original genotype-phenotype association; blue circles show the distribution of associations accounting for any relatedness in the DGRP. (a) Night sleep. (b) Day sleep. (c) Night bout number. (d) Day bout number. (e) Night average bout length. (f) Day average bout length. (g) Waking activity. (h) Night sleep CV_E. (i) Day sleep CV_E. (j) Night bout number CV_E. (k) Day bout number CV_E. (l) Night average bout lenght CV_E. (m) Day average bout number CV_E. (n) Waking activity CV_E.
