# Supplementary material for: Genome-wide association study of sleep in Drosophila melanogaster
Source: BMC Genomics. 2013 Apr 25;14:281. doi: 10.1186/1471-2164-14-281 (PMC3644253; doi:10.1186/1471-2164-14-281)
Supplement: Additional file 7 — P-value histograms for males. [file 1471-2164-14-281-S7.pdf]

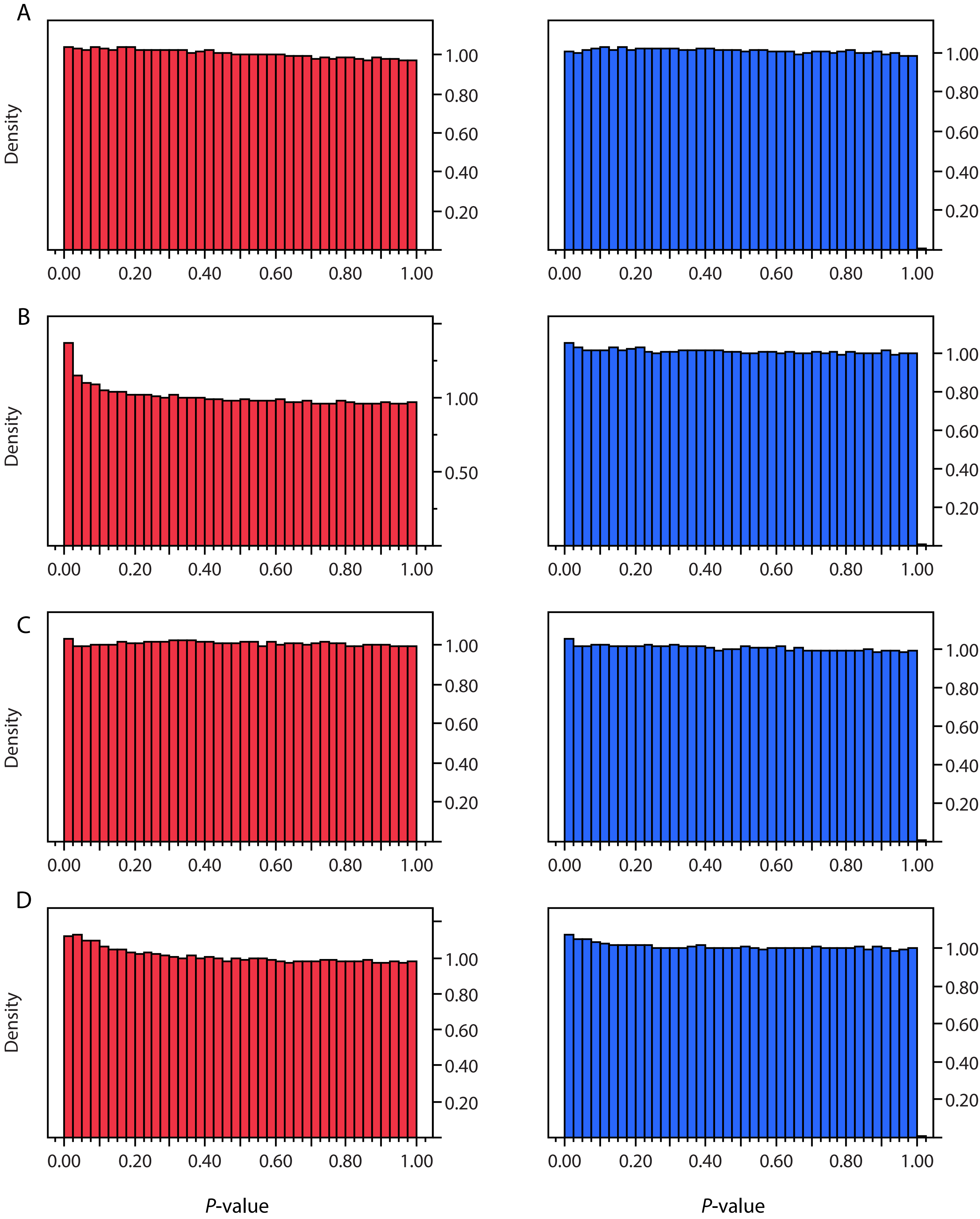

A
B
C
D
Density
1.00
0.80
0.60
0.40
0.20
0.50
0.00
P-value

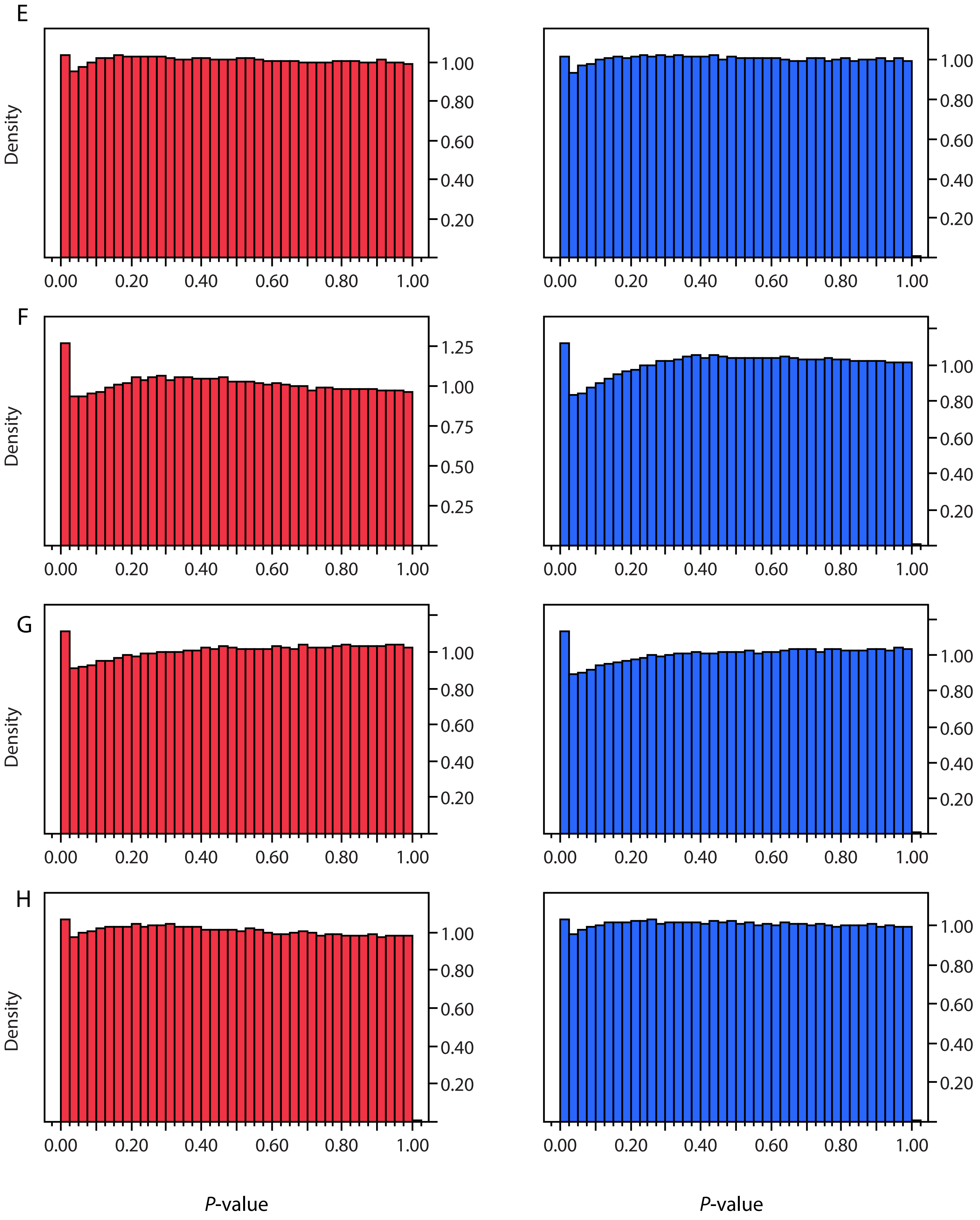

E
F
G
H
Density
0.00
0.20
0.40
0.60
0.80
1.00
0.25
0.50
0.75
1.25
P-value

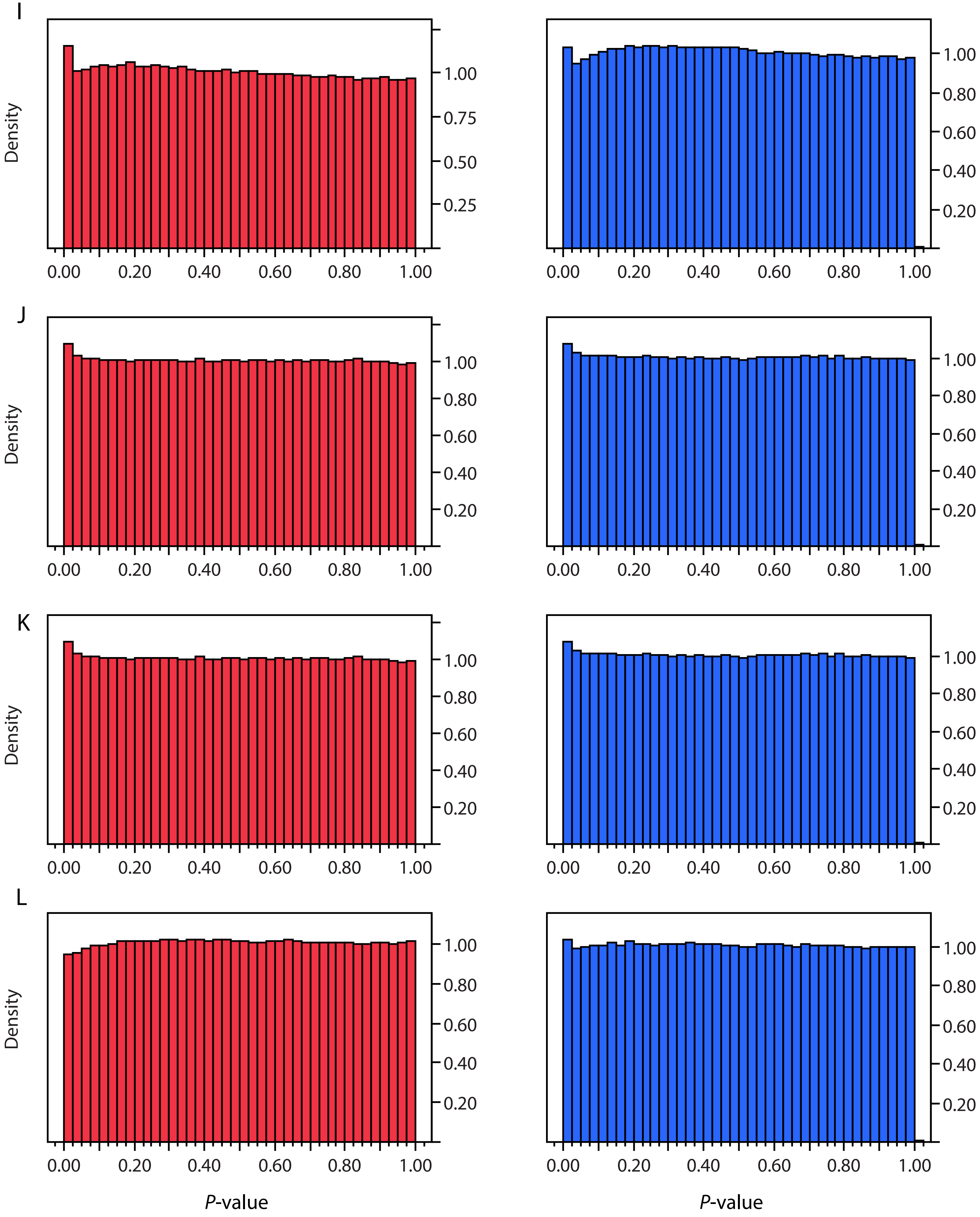
I
J
K
L
Density
P-value
0.00
0.20
0.40
0.60
0.80
1.00
0.25
0.50
0.75

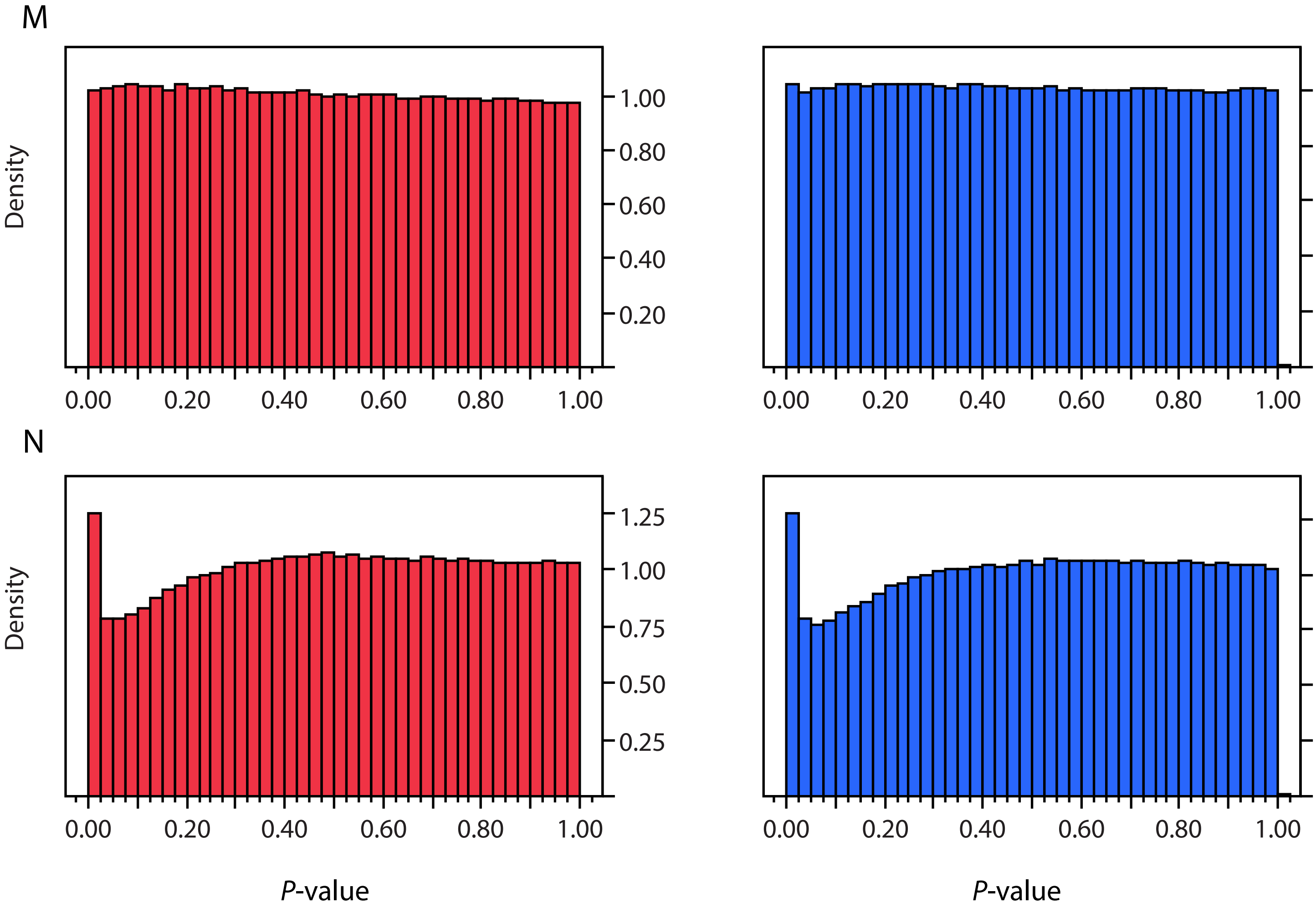

Additional file 7.*P*-value histograms for males.
The red histogram shows the *P*-value distribution of the original genotype-phenotype association; for each trait; the blue histogram shows the *P*-value distribution of associations accounting for any relatedness in the DGRP. (a) Night sleep. (b) Day sleep. (c) Night bout number. (d) Day bout number. (e) Night average bout length. (f) Day average bout length. (g) Waking activity. (h) Night sleep CV_E. (i) Day sleep CV_E. (j) Night bout number CV_E. (k) Day bout number CV_E. (l) Night average bout lenght CV_E. (m) Day average bout number CV_E. (n) Waking activity CV_E.
