# Supplementary material for: Genome-wide association study of sleep in Drosophila melanogaster
Source: BMC Genomics. 2013 Apr 25;14:281. doi: 10.1186/1471-2164-14-281 (PMC3644253; doi:10.1186/1471-2164-14-281)
Supplement: Additional file 11 — Minor allele frequency versus normalized effect size. [file 1471-2164-14-281-S11.pdf]

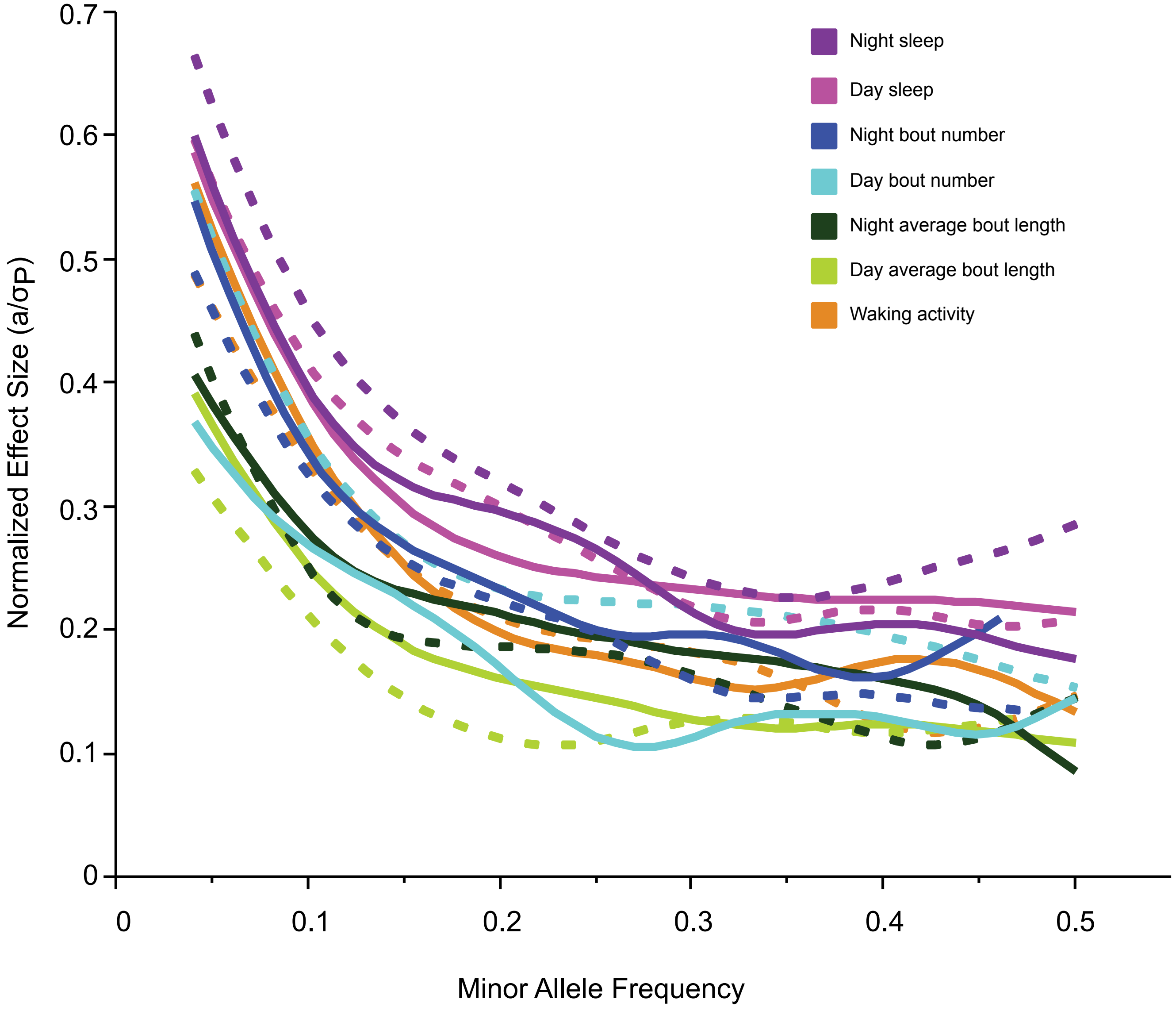

Additional file 11. Minor allele frequency vs. normalized effect size. Solid and dashed lines represent the trait mean and CV_E, respectively.
