# Supplementary material for: Genome-wide association study of sleep in Drosophila melanogaster
Source: BMC Genomics. 2013 Apr 25;14:281. doi: 10.1186/1471-2164-14-281 (PMC3644253; doi:10.1186/1471-2164-14-281)
Supplement: Additional file 17 — Pleiotropic effects in sleep candidate genes. [file 1471-2164-14-281-S17.pdf]

Additional file 17. Pleiotropic effects in candidate genes

An asterisk (*) indicates a trait that does not have a significant genetic correlation with the trait(s) in which the gene was identified.

Gene	Trait identified in GWAS	Trait pleiotropic effect observed
brinker	Night sleep<br>Night sleep CV_E	Day bout number*<br>Day sleep<br>Night bout number<br>Waking activity*<br>Day avg. bout length<br>Day bout number CV_E<br>Waking activity CV_E<br>Night avg. bout length CV_E
CG11163	Night sleep CV_E	Day bout number*<br>Day sleep<br>Night bout number<br>Waking activity*<br>Day avg. bout length<br>Night avg. bout length<br>Day bout number CV_E
CG12163	Night sleep CV_E	Day bout number*<br>Day sleep<br>Night bout number<br>Waking activity*<br>Day avg. bout length<br>Night avg. bout length
CG14545	Night sleep<br>Night sleep CV_E	Day bout number*<br>Day sleep<br>Waking activity*
fz	Night sleep CV_E<br>Waking activity CV_E	Day bout number*<br>Day sleep<br>Night bout number<br>Night sleep<br>Day avg. bout length<br>Night avg. bout length<br>Day bout number CV_E<br>Night bout number CV_E<br>Day avg. bout length CV_E<br>Night avg. bout length CV_E

Hey	Night sleep CV_E	Day bout number*<br>Day sleep<br>Night bout number<br>Night sleep<br>Waking activity*<br>Day avg. bout length<br>Night avg. bout length
scrib	Night sleep<br>Night sleep CV_E<br>Waking activity CV_E	Day bout number*<br>Day sleep<br>Night bout number<br>Waking activity<br>Day avg. bout length<br>Night avg. bout length<br>Day bout number CV_E
tkv	Day bout number CV_E<br>Waking activity CV_E	Day bout number<br>Night bout number<br>Night sleep<br>Waking activity<br>Day avg. bout length<br>Night avg. bout length<br>Night sleep CV_E
Ubx	Day avg. bout length<br>Day bout number CV_E<br>Waking activity CV_E	Day bout number<br>Day sleep<br>Night sleep<br>Waking activity<br>Day sleep CV_E<br>Night avg. bout length CV_E
unc-119	Night sleep<br>Night sleep CV_E	Day bout number*<br>Day sleep<br>Night bout number<br>Waking activity*<br>Day avg. bout length<br>Night avg. bout length<br>Waking activity CV_E
Vmat	Night sleep<br>Night sleep CV_E<br>Day avg. bout length	Night bout number<br>Waking activity<br>Night avg. bout length
